# Impact of X-linked hypophosphatemic rickets/osteomalacia on health and quality of life: baseline data from the SUNFLOWER longitudinal, observational cohort study

**DOI:** 10.1093/jbmrpl/ziae118

**Published:** 2024-09-10

**Authors:** Noriyuki Namba, Nobuaki Ito, Toshimi Michigami, Hee Gyung Kang, Takuo Kubota, Osamu Miyazaki, Ayumi Shintani, Daijiro Kabata, Yayoi Nishida, Seiji Fukumoto, Keiichi Ozono

**Affiliations:** Division of Pediatrics and Perinatology, Tottori University Faculty of Medicine, Tottori 683-8504, Japan; Division of Therapeutic Development for Intractable Bone Diseases, Graduate School of Medicine and Faculty of Medicine, The University of Tokyo, Tokyo 113-0033, Japan; Department of Bone and Mineral Research, Osaka Women’s and Children’s Hospital, Osaka Prefectural Hospital Organization, Osaka 594-1101, Japan; Division of Pediatric Nephrology, Department of Pediatrics, Seoul National University Children’s Hospital, Seoul 03080, South Korea; Department of Pediatrics, Osaka University Graduate School of Medicine, Suita 565-0871, Japan; Department of Radiology, National Center for Child Health and Development, Tokyo 157-8535, Japan; Department of Medical Statistics, Graduate School of Medicine, Osaka Metropolitan University, Osaka 545-8585, Japan; Department of Medical Statistics, Graduate School of Medicine, Osaka Metropolitan University, Osaka 545-8585, Japan; Center for Mathematical and Data Sciences, Kobe University, Hyogo 657-8501, Japan; Medical Affairs Department, Kyowa Kirin Co., Ltd., Tokyo 100-0004, Japan; Tamaki-Aozora Hospital, Tokushima 779-3125, Japan; Center for Promoting Treatment of Intractable Diseases, ISEIKAI International General Hospital, Osaka 530-0052, Japan

**Keywords:** clinical trials, disorders of calcium/phosphate metabolism, health economics, osteomalacia and rickets, Pth/vit d/fgf23

## Abstract

The SUNFLOWER study was initiated in Japan and South Korea to clarify the course of X-linked hypophosphatemic rickets/osteomalacia (XLH); delineate its physical, mental, and financial burdens; and collect information on treatment. Here, we report cross-sectional data at the time of patient enrollment to better understand the real-world management and complications in patients with XLH and examine the effect of XLH on quality of life (QOL). This is an ongoing, longitudinal, observational cohort study of patients with a diagnosis of XLH. Data from 147 patients (118 in Japan and 29 in South Korea) were evaluated. In total, 77 children (mean age, 9.7 yr; 67.5% female) and 70 adults (mean age, 37.6 yr; 65.7% female) were enrolled. *PHEX* gene mutations were confirmed in 46/77 (59.7%) children and 37/70 (52.9%) adults. Most patients in both age groups were receiving a combination of phosphate and active vitamin D at baseline. The mean height Z-score was −2.21 among adults (male: −2.34; female: −2.14). The mean Rickets Severity Score in children was 1.62. Whereas children appeared to have low pain levels (mean revised faces pain scale score, 1.3), adults reported mild-to-moderate pain (mean Brief Pain Inventory pain severity, 2.02). Mean QOL in children (assessed using the 10-item short-form health survey for children) was low, with a score below normative level for physical functioning. In adults, results from the Western Ontario and McMaster Universities osteoarthritis index indicated the presence of pain, stiffness, and decreased physical function. The respective mean total days/year of work/school non-attendance due to symptoms/complications and management of XLH were 0.7 and 3.0 among adults, and 6.4 and 6.1 among children. Our findings reconfirmed a relationship between disease and QOL in patients with XLH. We anticipate that these data will be important in enabling clinicians to understand the daily reality of patients with XLH.

## Introduction

X-linked hypophosphatemic rickets/osteomalacia (XLH) is a rare disease with an X-linked dominant inheritance pattern.^1^ XLH is caused by an excess of fibroblast growth factor 23 (FGF23)^2^^,^^3^ due to inactivating mutations of *phosphate-regulating gene with homologies to endopeptidases on the X chromosome* (*PHEX*).^1^ Patients with XLH exhibit lifelong persistent hypophosphatemia, manifesting as growth retardation during childhood and causing ongoing medical issues related to bone deformities.^4–7^ The reported incidence of XLH is estimated to be 1 per 20 000 to 60 000 live births.^8–10^

FGF23 is a central regulator of serum phosphate concentration and causes a decrease in serum phosphate concentration via two mechanisms.^10^^,^^11^ The first mechanism is the inhibition of phosphate reabsorption in the renal proximal tubule. The second mechanism is decreased intestinal absorption of phosphate due to reduced concentration of 1,25-dihydroxyvitamin D, which is caused by the inhibition of renal 1-α-hydroxylase activity and the acceleration of 24-hydroxylase activity.

Because of hypophosphatemia, many patients with XLH develop rickets in childhood, producing the characteristic manifestations of skeletal abnormalities (such as bowing leg) and failure to thrive, and resulting in short stature.^12^^,^^13^ Growth plate closure and cessation of growth alter the symptoms observed in adults. Adult patients with XLH are at increased risk of developing bone pain and fractures, joint abnormalities, enthesopathy, osteophytes, muscular weakness, and ossification of the spinal ligament.^5^ Patients may also require surgical correction for the limb deformities incurred during childhood.^6^^,^^14^^,^^15^

Several observational studies have reported the impact of XLH on patients’ daily life, health, and functioning. These include the effects of XLH on growth of children,^15^^,^^16^ the relationship between childhood treatment and adult complications,^17^ the reduction in quality of life (QOL) in adult patients with XLH compared with healthy subjects and individuals with other bone diseases,^18^^,^^19^ and the risk of additional medical complications such as early onset of hypertension.^20^ However, because XLH is a rare disease, the number of patients in each study was limited, hampering the ability to draw definitive conclusions or gain a consensus on the optimal treatment.

Notably, there are currently no global unified guidelines for the identification and management of XLH. Individual countries and regions including the United States,^5^ Europe,^21^ and Japan,^22^ have their own recommendations, guidelines, or diagnostic criteria; thus, diagnostic and treatment policies may differ depending on the medical institution or geographic area.

Large-scale and long-term observational studies are necessary to allow physicians to fully understand the disease course of XLH and to unify treatment policies. In 2018, the longitudinal, observational SUNFLOWER (Study of longitUdinal observatioN For patients with X-Linked hypOphosphatemic rickets/osteomalacia in collaboration With Asian partnERs) study was initiated in Japan and South Korea, with the aims of clarifying the course of the disease; delineating the physical, mental, and financial burden; and collecting information on treatment.^23^ In this article, we report patient information at the time of enrollment into the SUNFLOWER study, for the purpose of understanding real-world management and complications in patients with XLH, and examining the effect of XLH on QOL.

## Patients and methods

### Patients

Full details of the SUNFLOWER study protocol, including patient eligibility, have been reported.^23^ In brief, the inclusion criteria were patients with a diagnosis of XLH who had a documented *PHEX* gene mutation and/or a first-degree relative with a documented *PHEX* gene mutation and/or a documented intact FGF23 level of more than 30 pg/mL; current or previous physical examination findings or laboratory findings of rickets/osteomalacia; and provision of written informed consent by all adult patients or additional written consent from the parents or legal representatives of patients aged *<*20 yr. The diagnosis of XLH was based on the decision of individual physicians; however, documentation of gene mutation or FGF23 levels was necessary to confirm the diagnosis for this study. The cut-off level for FGF23 was selected based on previously published data as the value necessary to diagnose FGF23-related hypophosphatemia.^24^ No specific criteria were set for specific XLH stage or severity, in order to obtain general real-world data on XLH.

Exclusion criteria included participation in another clinical study at the time of informed consent, and any patient whose participation in the study was considered inappropriate or unsafe by the investigator. Patients were permitted to enroll into the SUNFLOWER study after the completion of other clinical studies.

### Study design

The SUNFLOWER study is an ongoing, longitudinal, observational cohort study of patients with XLH; enrollment began on April 1, 2018 and closed on April 30, 2022. For this analysis of baseline data, the information on patients enrolled between April 1, 2018 and December 31, 2019 was evaluated. Patients in this analysis were enrolled from 20 medical institutions (17 in Japan and 3 in South Korea). The study is registered under the identification numbers NCT03745521 and UMIN000031605.

The study is being conducted in compliance with the most recent version of the Declaration of Helsinki and all applicable national regulations in Japan (including the Ethical Guidelines for Medical and Health Research Involving Human Subjects) and South Korea (including local regulations and guidelines). The protocol and the informed consent documentation were approved by the Ethics Committee of Osaka University Graduate School of Medicine, the Ethics Committee of Kyowa Kirin, and the Ethics Committee of each participating medical institution.

### Assessments

Study assessments and measurements implemented at baseline were patient characteristics; height and body weight; Tanner stage (for patients aged *<*18 yr); blood pressure; radiography of the bones; renal ultrasonography; dental assessment; use of drugs to treat XLH and its complications; fractures confirmed by medical records; complications confirmed by medical records; surgery for the underlying disease and its complications (eg, craniotomy/craniectomy, osteotomy, epiphysiodesis, joint replacement, fracture repair, ligament/cartilage repair, bone shaving/bone spur removal, tendon resection/reimplantation, and spinal decompression); laboratory assessments (blood and spot urine); motor function; QOL; and loss of working/schooling. Random blood and urine sampling was performed at least 4 h after a meal or intake of a phosphate preparation in principle. Radiographic assessment to evaluate Rickets Severity Score (RSS) and lower limb deformity for patients aged <18 yr included anteroposterior views of both knees and posteroanterior views of both wrists. Intact FGF23 levels (Determiner CL FGF23, Minaris Medical Co., Ltd., Tokyo, Japan) at baseline correspond to those measured after conventional therapy. Nephrocalcinosis was assessed by renal ultrasound using the following 5-grade system: Grade 0: normal, Grade 1: mild echogenicity around renal pyramid borders, Grade 2: severe echogenicity around renal pyramid borders and minor echogenicity of entire renal pyramids, Grade 3: uniformly severe echogenicity of entire renal pyramids, and Grade 4: calculus (echogenicity indicating a solitary lesion at the tip of renal pyramids). To evaluate lower limb deformity, the mechanical axis deviation was measured; the limb with the greater deformity when comparing the left and right side was used. Motor function assessments were conducted for patients aged ≥5 to *<*18 yr using the 6-min walk test (6MWT). For patients aged ≥18 yr, measures included the Timed Up and Go Test (TUGT) and grip strength measurement (with both hands, in a sitting position). QOL and loss of working/schooling were evaluated using patient-reported outcome (PRO) measures including the revised faces pain scale (FPS-R; *<*18 yr),^25^ the 10-item short-form health survey for children (SF-10; *<*18 yr),^26^ the brief pain inventory (BPI; ≥18 yr),^27^ and the Western Ontario and McMaster Universities osteoarthritis index (WOMAC; ≥18 yr).^28^ Specifically, missed work/school days/year due to symptoms/complications of XLH or due to XLH treatment were counted as a measure of job/study participation.

The aim of the data analyses was to define the characteristics of patients with XLH using comorbidity rates by age; to clarify the physical and mental burdens on the patient; and to evaluate the effectiveness and safety of conventional therapy for XLH.

PRO scores were used to assess pain, disability, and health-related QOL in children and adults. The FPS-R was used to assess pain intensity in children aged 5-17 yr. It was adapted from the original FPS to allow scoring of pain sensation on a 0-10 metric, with a higher score indicating higher pain intensity. The BPI was used to assess pain severity and interference with daily life in adults. Pain intensity was measured in four categories (worst, least, average, and current), while pain interference was measured in seven categories (mood, work, general activity, walking, relationships, enjoyment of life, and sleep); each category was rated on a scale from 0 to 10, with a higher score indicating either higher pain severity or increased impact on daily life.

The SF-10 was used to assess general health-related QOL of children aged 5-17 yr. It consists of the physical summary score encompassing five items related to physical activity, energy, movement, and pain. It also consists of the psychosocial summary score encompassing five items related to friendships, social participation, and emotional issues. SF-10 scores were transformed using norm-based scoring^29^; this standardized the values with respect to United States population norms (mean value, 50; SD, 10).

WOMAC scores were used to assess joint pain, joint stiffness, and physical function among patients aged ≥18 yr. WOMAC measures five items for pain (score range 0-20), two for stiffness (score range 0-8), and 17 for functional limitation (score range 0-68); a higher score correlates with poorer function. WOMAC scores were normalized to a 0-100 metric representing the percent of a maximum score.

### Statistical methods

The target sample size of this study is 240 patients, consisting of 180 patients in Japan and 60 patients in South Korea. Continuous variables were described using summary statistics (number of patients, mean, SD, SE, minimum, maximum, and interquartile range). Categorical data were summarized using frequency or proportion of patients. The relationship between the patient’s age and the standardized height (Z-score) was assessed using a non-linear regression model in all patients as well as subpopulations among male and female patients.

To examine the relationships between PRO scores and complication status, we performed analyses with proportional-odds logistic regression models for each complication, separately. We used the SF-10 and FPS-R for patients aged *<*18 yr, and the BPI and school/employment score for WOMAC for patients aged ≥18 yr. Furthermore, we examined the relationship between each pair of medical complications using binary logistic regression models. The strength of the association between each pair of factors was evaluated by the logarithmic odds ratio (LOR). In addition, we examined the relationship between working/schooling scores and each PRO using the proportional-odds logistic regression model. For subgroup estimation, we performed similar analyses as those described above among the subpopulations of male and female patients. In the above regression models, all missing values were imputed using a multiple imputation approach based on the predictive mean matching method.

We employed a two-sided 5% significance level for all statistical hypothesis testing. All statistical analyses were performed using SAS software, version 9.4 (SAS Institute Inc., Cary, NC, USA) and R version 4.0.2 (R Foundation for Statistical Computing, Vienna, Austria), and *p*-value adjustments for multiplicity were not performed.

**Table 1:** Baseline patient characteristics.

Characteristics	Children (*n =* 77)	Adults (*n =* 70)
**Age, yr, (range)**	9.7 ± 4.9 (0–17)	37.6 ± 15.8 (18–78)
**Sex, female (%)**	52 (67.5)	46 (65.7)
**Geographic region**		
**Japan**	62 (80.5)	56 (80.0)
**South Korea**	15 (19.5)	14 (20.0)
**XLH diagnosis**		
***PHEX* gene mutation**	46 (59.7)	37 (52.9)
** Family history**	32 (41.6)	32 (45.7)
** FGF23 level > 30 pg/mL**	49 (63.6)	50 (71.4)
**Medications**		
**Both phosphate and active vitamin D treatment**	77 (100)	57 (81.4)
**Phosphate treatment only**	0 (0)	1 (1.4)
**Active vitamin D treatment only**	0 (0)	10 (14.3)
**No medication**	0 (0)	2 (2.9)
**Height, Z-score**	−1.97 ± 1.39	−2.21 ± 1.51
**Males**	−2.39 ± 1.70	−2.34 ± 1.42
**Females**	−1.77 ± 1.18	−2.14 ± 1.57
**Weight, Z-score**	−0.45 ± 0.96	−0.49 ± 1.53
**Males**	−0.77 ± 0.80	−0.30 ± 1.67
**Females**	−0.29 ± 1.00	−0.60 ± 1.46
**BMI, Z-score**	0.79 ± 0.95	0.67 ± 1.13
**Males**	0.81 ± 0.90	0.85 ± 1.22
**Females**	0.79 ± 0.98	0.57 ± 1.09
**Blood pressure**		
**Systolic, mmHg**	105.7 ± 11.8	121.3 ± 16.4
**Diastolic, mmHg**	62.1 ± 9.8	73.6 ± 14.6
**Laboratory parameters** ** (Reference value)**		
**Serum phosphate, mg/dL** ** (Adults: 2.4–4.3** ** Children: Age-dependent normal range^a^)**	2.81 ± 0.67 (1.6–4.8)	2.22 ± 0.45 (1.3–3.3)
**TmP/GFR, mg/dL** ** (Adults: 2.3–4.3** ** Children: 1–10 yr old: 5.31 ± 0.4** ** 10–15 yr old: 4.52 ± 1.1)**	2.13 ± 0.59	1.65 ± 0.51
**Serum 1,25(OH)2D, pg/mL** **(Adults: 20–60** **Children: 20–70)**	55.3 ± 24.9	42.5 ± 23.5
**Serum 25(OH)D, ng/mL** **(Deficiency: <20)**	21.2 ± 5.6	19.8 ± 14.6
**Serum ALP (IFCC), IU/L** **(Children: Age-dependent normal range^a^)**	439.2 ± 222.0	–
**Serum BALP, ug/L** **(Males: 3.7–20.9** **Females: Premenopausal 2.9–14.5** **Postmenopausal 3.8–22.6)**	–	30.4 ± 23.1
**Serum calcium, mg/dL** ** (Adults: 8.5–10.2** ** Children: Age-dependent normal range^a^)**	9.44 ± 0.37	9.34 ± 0.43
**Serum iPTH, pg/mL** ** (10–65)**	56.3 ± 34.9	89.4 ± 107.5
**Serum creatinine, mg/mL** ** (Male Adults: 0.61–1.04** ** Female Adults: 0.47–0.79** ** Children: Age-dependent normal range^a^)**	0.40 ± 0.14	0.66 ± 0.42
**eGFR, mL/min/1.73m^2^** ** (Adults: >60** ** Children: 1–1.5 yr old: 83.3–132.6** ** 1.5–16 yr old: 83.5–156.7)**	117.2 ± 23.1	110.4 ± 36.6
**Intact FGF23, pg/dL** ** (FGF-related hypophosphatemia: >30)**	268.5 ± 314.0	400.0 ± 654.7

Data are mean ± SD, (min–max), or *n* (%).

^a^Age-dependent normal range. Normative values by age are available in references^30^^,^^31^ and Table S4.

Abbreviations: ALP = alkaline phosphatase; BALP = bone-specific alkaline phosphatase; FGF = fibroblast growth factor; eGFR = estimated glomerular filtration rate; iPTH = intact parathyroid hormone; NA = not applicable; TmP/GFR = ratio of tubular maximum reabsorption rate of phosphate to glomerular filtration rate; XLH = X-linked hypophosphatemic rickets/osteomalacia.

**Figure 1:**
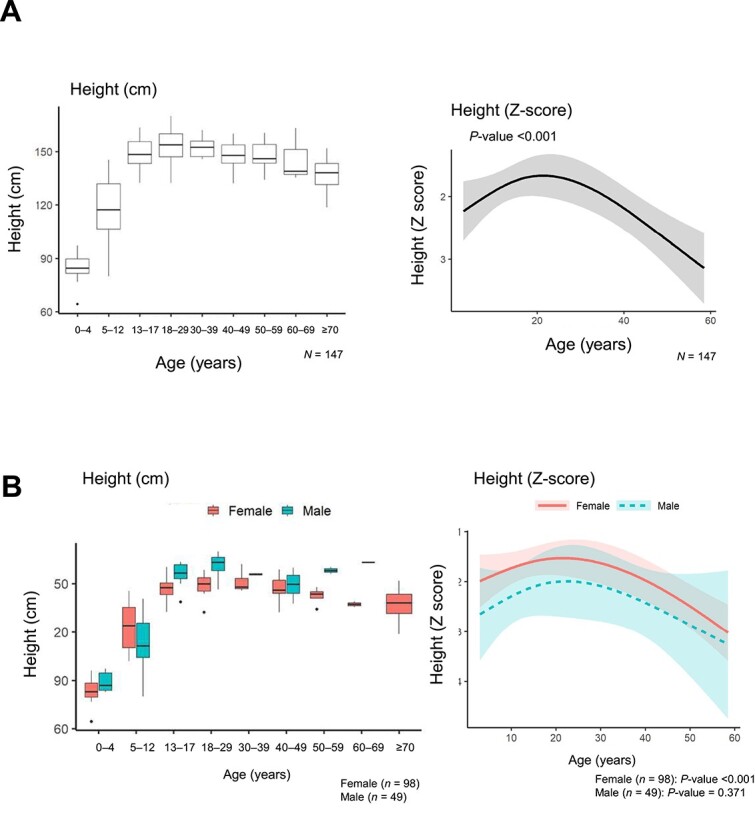
Height and height Z-score at enrollment versus age for all patients (A) and according to sex (B). Height is shown in the box-plot and height Z-scores are shown in regression models.

## Results

### Patients

In this analysis, data from 147 patients (118 in Japan and 29 in South Korea) were evaluated. Key baseline patient characteristics are shown in Table 1. A total of 77 children were enrolled; their mean ± SD age was 9.7 ± 4.9 yr, and 52/77 (67.5%) were females. There were 70 adults; the mean age was 37.6 ± 15.8 yr and 46/70 (65.7%) were females. *PHEX* gene mutations were confirmed in 46/77 children (59.7%) and 37/70 adults (52.9%). The proportion of patients with a family history of XLH was 41.6% (32/77) among the children and 45.7% (32/70) among the adults. The proportion of patients diagnosed based on FGF23 level >30 pg/mL was 63.6% (49/77) among children and 71.4% (50/70) among adults. The majority of patients in both age groups were receiving phosphate and active vitamin D treatment at baseline (77/77 children [100%]; 57/70 adults [81.4%]). All children received both phosphate and active vitamin D treatment. While in adults, 1/70 (1.4%) received phosphate treatment only, 10/70 (14.3%) received active vitamin D treatment only, and 2/70 (2.9%) received no medication. The mean ± SD age at treatment initiation was 3.8 ± 3.4 yr among children and 21.2 ± 16.7 yr among adults. The mean ± SD height Z-score was −1.97 ± 1.39 among children and − 2.21 ± 1.51 among adults (adult male patients: −2.34 ± 1.42 and adult female patients: −2.14 ± 1.57). Height and height Z-scores at the time of enrollment plotted according to age are shown for all patients and by sex in Figure 1A and B, respectively. The mean ± SD weight Z-score was −0.45 ± 0.96 among children and −0.49 ± 1.53 among adults (adult male patients: −0.30 ± 1.67 and adult female patients: −0.60 ± 1.46). The mean ± SD BMI Z-score was 0.79 ± 0.95 among children and 0.67 ± 1.13 among adults (adult male patients: 0.85 ± 1.22 and adult female patients: 0.57 ± 1.09). Laboratory parameters at baseline are summarized in Table 1. For the children, the mean ± SD values for serum parameters were as follows: phosphate, 2.81 ± 0.67 mg/dL; 1,25(OH)2D, 55.3 ± 24.9 pg/mL; 25(OH)D, 21.2 ± 5.6 ng/mL; calcium, 9.44 ± 0.37 mg/dL; and intact FGF23, 268.5 ± 314.0 pg/dL. In addition, the ratio of tubular maximum reabsorption rate of phosphate to glomerular filtration rate (TmP/GFR) for children was 2.13 ± 0.59 mg/dL. For the adults, the mean ± SD values for serum parameters were as follows: phosphate, 2.22 ± 0.45 mg/dL; 1,25(OH)2D, 42.5 ± 23.5 pg/mL; 25 (OH)D, 19.8 ± 14.6 ng/mL; bone-specific alkaline phosphatase, 30.4 ± 23.1 ug/L; calcium, 9.34 ± 0.43 mg/dL; and intact FGF23, 400.0 ± 654.7 pg/dL. In addition, TmP/GFR for adults was 1.65 ± 0.51 mg/dL.

### Physical and functional assessments

The results of physical and functional assessments are shown in Table 2. The mean ± SD RSS and mechanical axis deviation in children were 1.62 ± 1.11 (males: 2.36 ± 1.13 and females: 1.10 ± 0.76) and 20.3 ± 13.6 mm, respectively. The mean ± SD 6MWT distance was 439.6 ± 83.3 m in children (males: 449.4 ± 89.1 m and females: 435.2 ± 81.3 m). The mean ± SD TUGT time was 12.8 ± 10.6 s in adults (males: 12.6 ± 5.5 s and females: 12.9 ± 12.6 s). The mean ± SD grip strength was 24.7 ± 8.1 kg in adults (males: 31.7 ± 7.5 kg and females: 21.0 ± 5.6 kg).

### Comorbidities

Comorbidity rates, confirmed by medical records or ultrasound, of patients with XLH by age group are shown in Table 3. Among adults, 7.1% (5/70) and 4.3% (3/70) had stage 3 and stage 4 renal dysfunction, respectively. By ultrasound, the proportion of children with nephrocalcinosis Grade 0 was 37.7%; Grade 1, 6.5%; Grade 2, 7.8%; and Grade 3, 1.3%. By ultrasound, the proportion of adults with nephrocalcinosis Grade 0 was 30.0%; Grade 1, 12.9%; Grade 2, 15.7%; Grade 3, 8.6%; and Grade 4, 5.7%. The proportions of patients with a diagnosis of nephrocalcinosis were 26.0% (20/77) among the children and 41.4% (29/70) among adults. In children and adults, the proportions of patients with comorbidities were as follows: nephrolithiasis, 2.6% (2/77) and 10.0% (7/70); hypercalciuria, 5.2% (4/77) and 11.4% (8/70); hypercalcemia, 3.9% (3/77) and 7.1% (5/70); and hyperparathyroidism, 5.2% (4/77) and 32.9% (23/70), respectively. Among children, increased intracranial pressure was reported in one patient (1.3%).

**Table 2:** Physical and functional assessments.

	Children		Adults	
	*n*	Mean ± SD	*n*	Mean ± SD
**Rickets severity score (0–10)**	34	1.62 ± 1.11	–	–
**Males**	–	2.36 ± 1.13	–	–
**Females**	–	1.10 ± 0.76	–	–
**Lower limb deformity**	–	–	–	–
**MAD, mm**	50	20.3 ± 13.6	–	–
**Physical function**	–	–	–	–
**6MWT, m**	61	439.6 ± 83.3	–	–
**Males**	–	449.4 ± 89.1	–	–
**Females**	–	435.2 ± 81.3	–	–
**TUGT, s**	–	–	67	12.8 ± 10.6
**Males**	–	–	–	12.6 ± 5.5
**Females**	–	–	–	12.9 ± 12.6
**Grip strength, total, kg**	–	–	69	24.7 ± 8.1
**Males**	–	–	24	31.7 ± 7.5
**Females**	–	–	45	21.0 ± 5.6

Abbreviations: 6MWT = 6-min walk test; MAD = mechanical axis deviation; TUGT = Timed Up and Go Test; XLH = X-linked hypophosphatemic rickets/osteomalacia.

**Table 3:** Baseline comorbidity rates of patients with XLH by age group.

Variables	Children	Adults
	*n =* 77	*n =* 70
	*n* (%)	*n* (%)
**Iatrogenic**		
**Renal dysfunction**		
**Stage 3**	0 (0)	5 (7.1)
**Stage 4**	0 (0)	3 (4.3)
**Nephrocalcinosis (ultrasound)**		
**Grade 0**	29 (37.7)	21 (30.0)
**Grade 1**	5 (6.5)	9 (12.9)
**Grade 2**	6 (7.8)	11 (15.7)
**Grade 3**	1 (1.3)	6 (8.6)
**Grade 4**	0 (0)	4 (5.7)
**Nephrolithiasis**	2 (2.6)	7 (10.0)
**Hypercalciuria**	4 (5.2)	8 (11.4)
**Hypercalcemia**	3 (3.9)	5 (7.1)
**Hyperparathyroidism**	4 (5.2)	23 (32.9)
**Hypertension**	0 (0)	21 (30.0)
**Bone deformity and related symptoms**		
**Femoral curvature**	21 (27.3)	22 (31.4)
**Tibial/fibular (lower leg) curvature**	27 (35.1)	23 (32.9)
**Genu varum (bowleg)**	52 (67.5)	34 (48.6)
**Pigeon-toed gait**	19 (24.7)	15 (21.4)
**Genu valgum (knock-knees)**	15 (19.5)	12 (17.1)
**Abnormal gait/running**	30 (39.0)	22 (31.4)
**Leg length discrepancy**	8 (10.4)	11 (15.7)
**Coxa vara**	4 (5.2)	1 (1.4)
**Ectopic ossification and related symptoms**		
**Osteophyte**	2 (2.6)	23 (32.9)
**Enthesopathy**	0 (0)	19 (27.1)
**Spinal canal stenosis**	0 (0)	5 (7.1)
**OPLL**	0 (0)	10 (14.3)
**OALL**	0 (0)	0 (0)
**OLF**	0 (0)	12 (17.4)
**Joint stiffness**	0 (0)	2 (2.9)
**Osteoarthritis**	1 (1.3)	4 (5.7)
**Pain**		
**Bone pain**	7 (9.1)	17 (24.3)
**Joint pain**	19 (24.7)	36 (51.4)
**Bone fracture**		
**Bone fracture**	3 (3.9)	24 (34.3)
**Surgery for bone fracture**	0 (0)	8 (11.4)
**Surgery**	18 (23.4)	37 (52.9)
**Dental problems**	15 (19.5)	37 (52.9)
**Others**		
**Deafness**	0 (0)	4 (5.7)
**Tinnitus**	0 (0)	6 (8.6)
**Dizziness**	0 (0)	5 (7.1)
**Chiari malformation**	0 (0)	0 (0)
**Syringomyelia**	0 (0)	0 (0)
**Craniosynostosis**	1 (1.3)	0 (0)
**Increased intracranial pressure**	1 (1.3)	0 (0)

Abbreviations: OALL = ossification of the anterior longitudinal ligament; OPLL = ossification of the posterior longitudinal ligament; OLF = ossification of the ligamentum flavum.

The most frequent bone deformities and related symptoms among children and adults were genu varum (bow leg) (67.5% [52/77] and 48.6% [34/70]), genu valgum (knock-knees) (19.5% [15/77] and 17.1% [12/70]), abnormal gait/running (39.0% [30/77] and 31.4% [22/70]), tibial/fibular (lower leg) curvature (35.1% [27/77] and 32.9% [23/70]), femoral curvature (27.3% [21/77] and 31.4% [22/70]), and pigeon-toed gait (24.7% [19/77] and 21.4% [15/70]), respectively. Regarding ectopic ossification (defined as a condition in which bone forms in tissues where it does not typically develop) and related symptoms, 2/77 children (2.6%) had osteophyte and the most frequent comorbidities among adults were osteophyte (32.9% [23/70]) and enthesopathy (27.1% [19/70]). Spinal canal stenosis was reported in five adults (7.1%). The proportions of patients among children and adults with bone pain were 9.1% (7/77) and 24.3% (17/70); joint pain, 24.7% (19/77) and 51.4% (36/70); bone fracture, 3.9% (3/77) and 34.3% (8/70); and dental problems, 19.5% (15/77) and 52.9% (37/77), respectively.

### QOL assessments

PROs and work/school status are summarized in Table 4. While children appeared to have low levels of pain (mean ± SE FPS-R of 1.3 ± 0.07), adults reported mild-to-moderate pain (mean ± SE BPI worst pain of 3.0 ± 0.35, pain severity of 2.02 ± 0.257, and pain interference 2.3 ± 0.034). QOL in children (assessed using the SF-10) appeared to be low, with mean ± SE scores below normative levels for physical functioning (47.0 ± 1.10). In adults, the results from the WOMAC index indicated the presence of pain (20.6 ± 2.55), stiffness (20.7 ± 2.99), and decreased physical function (18.2 ± 2.61).

**Table 4:** PRO and work/school status.

		Children		Adults	
PRO	Domain	*n*	Mean ± SE	*n*	Mean ± SE
**FPS-R**	Pain scale	61	1.3 ± 0.07	–	–
**BPI**	Worst pain	–	–	66	3 ± 0.35
	Pain severity	–	–	66	2.02 ± 0.26
	Pain interference	–	–	66	2.3 ± 0.34
**SF-10**	PHS-10 score (physical)	60	47 ± 1.10	–	–
	PSS-10 score (psychosocial)	60	53.5 ± 0.82	–	–
**WOMAC**	Subscale score, pain	–	–	64	20.6 ± 2.55
	Subscale score, stiffness	–	–	64	20.7 ± 2.99
	Subscale score, physical function	–	–	64	18.2 ± 2.61
**Work/school status**		***n***	**Mean ± SD**	***n***	**Mean ± SD**
**Work status**	Work non-attendance due to symptoms/complications of XLH, d/yr, range: 0–10	1	0	23	0.7 ± 2.2
	Work non-attendance due to management of XLH, d/yr, range: 0–12	1	0	23	3 ± 3.7
**School status**	School non-attendance due to symptoms/complications of XLH, d/yr, range: 0–240	40	6.4 ± 37.9	9	0.1 ± 0.3
	School non-attendance due to management of XLH, d/yr, range: 0–120	40	6.1 ± 18.7	9	3.1 ± 3.6

Abbreviations: BPI = brief pain inventory; FPS-R = revised faces pain scale; PRO = patient-reported outcome; SF-10 = 10-item short-form health survey; WOMAC = Western Ontario and McMaster Universities osteoarthritis index; XLH = X-linked hypophosphatemic rickets/osteomalacia.

Among adults, the mean ± SD total days/year of work non-attendance due to symptoms/complications of XLH was 0.7 ± 2.2 and mean ± SD total days/year of work non-attendance due to management of XLH was 3.0 ± 3.7. The mean ± SD total days/year of school non-attendance due to symptoms/complications was 0.1 ± 0.3 and mean ± SD total days/year of school non-attendance due to management of XLH was 3.1 ± 3.6. Among children, the mean ± SD days/year of school non-attendance due to symptoms/complications of XLH was 6.4 ± 37.9 and mean ± SD days/year of school non-attendance due to management of XLH was 6.1 ± 18.7.

### Relationships between baseline attributes and measures

**Figure 2:**
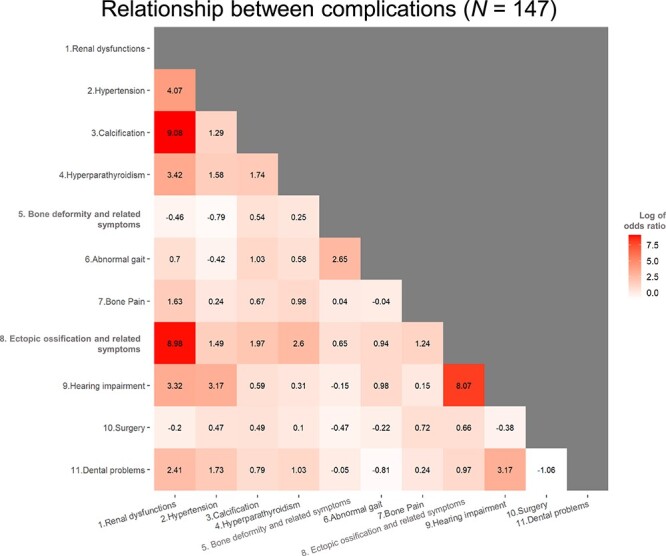
Unadjusted logistic regression showing relationships between comorbidities.

The relationships between comorbidities are shown in Figure 2. Important relationships (LOR > 2.0) were observed for renal dysfunction and calcification (9.08), ectopic ossification and related symptoms (8.98), hypertension (4.07), hyperparathyroidism (3.42), hearing impairment (3.32), and dental problems (2.41).

**Table 5:** Significant relationships between (A) QOL and comorbidity, (B) work/school status and comorbidity, and (C) work/school status and QOL.

A.														
Children		All (*N* = 77)				Subgroup analysis								
						Male (*n =* 25)				Female (*n =* 52)				
**Outcome**	Exposure	Diff (IQR)	Odds ratio (/IQR)	95% confidence interval	*p*	Diff (IQR)	Odds ratio (/IQR)	95% confidence interval	*p*	Diff (IQR)	Odds ratio (/IQR)	95% confidence interval	*p*	
**SF-10:** **PHS score**	Bone deformity and related symptoms	1	0.534	0.213–1.336	.18	1	1.733	0.3–10.004	.538	1	0.312	0.105–0.929	.036^*^	
	Ectopic ossification and related symptom	1	0.27	0.102–0.715	.008^*^	1	0.242	0.052–1.116	.069	1	0.21	0.06–0.735	.015^*^	
	Surgery	1	0.331	0.117–0.935	.037^*^	1	1.082	0.155–7.574	.937	1	0.1	0.022–0.445	.003^*^	
**SF-10: PSS score**	Hyperparathyroidism	1	5.325	1.187–23.891	.029^*^	1	1.566	0.085–28.982	.763	1	8.537	0.643–113.417	.104	
	Surgery	1	0.294	0.104–0.832	.021^*^	1	0.601	0.089–4.075	.603	1	0.158	0.036–0.695	.015^*^	
	6MWT	96	1.078	0.401–2.894	.882	104.5	3.968	1.055–14.919	.041^*^	98.25	0.792	0.402–1.562	.501	
**FPS-R**	Bone deformity and related symptoms	1	0.368	0.118–1.145	.084	1	0.061	0.005–0.786	.032^*^	1	0.826	0.224–3.047	.773	
**Adults**		**All (*N* = 70)**				**Subgroup analysis**								
						**Male (*n =* 24)**				**Female (*n =* 44)**				
**Outcome**	**Exposure**	**Diff (IQR)**	**Odds ratio** **(/IQR)**	**95% confidence interval**	***p***	**Diff (IQR)**	**Odds ratio** **(/IQR)**	**95% confidence interval**	***p***	**Diff (IQR)**	**Odds ratio** **(/IQR)**	**95% confidence interval**	***p***	
**BPI-pain severity (worst)**	Hypertension	1	3.267	1.231–8.672	.017^*^	1	0.897	0.218–3.691	.881	1	11.485	2.478–53.232	.002^*^	
	Height	11.4	0.779	0.472–1.285	.328	8.6	1.331	0.603–2.939	.48	8.2	0.562	0.321–0.984	.044^*^	
	Height (Z-score)	1.53	0.688	0.446–1.062	.091	1.478	1.332	0.603–2.944	.478	1.56	0.563	0.322–0.984	.044^*^	
	TUGT	3.385	1.237	1.082–1.416	.002^*^	4.863	2.276	1.182–4.382	.014^*^	3.05	1.19	1.037–1.366	.013^*^	
**BPI-pain severity (least)**	Renal dysfunction	1	3.078	0.797–11.882	.103	1	0.957	0.127–7.198	.966	1	6.885	1.073–44.154	.042^*^	
	Hypertension	1	3.209	1.179–8.732	.022^*^	1	1.152	0.264–5.034	.851	1	5.793	1.278–26.271	.023^*^	
	Calcification	1	2.911	1.101–7.698	.031^*^	1	1.679	0.382–7.367	.493	1	4.601	1.178–17.97	.028^*^	
	TUGT	3.385	1.373	1.104–1.708	.004^*^	4.863	1.378	0.735–2.584	.317	3.05	1.288	1.051–1.578	.015^*^	
	Grip strength	8	0.832	0.519–1.334	.446	9.5	0.268	0.081–0.881	.03^*^	8	0.653	0.262–1.627	.36	
**BPI-pain severity (average)**	Renal dysfunction	1	4.067	1.05–15.751	.042^*^	1	1.474	0.158–13.718	.733	1	7.644	1.321–44.221	.023^*^	
	Hypertension	1	4.787	1.782–12.861	.002^*^	1	1.167	0.28–4.86	.832	1	15.601	3.392–71.764	<.001^*^	
	Height	11.4	0.746	0.455–1.224	.246	8.6	0.933	0.42–2.072	.864	8.2	0.521	0.302–0.9	.019^*^	
	Height (Z-score)	1.53	0.64	0.411–0.997	.048^*^	1.478	0.934	0.42–2.076	.867	1.56	0.522	0.303–0.901	.019^*^	
	TUGT	3.385	1.218	1.06–1.4	.005^*^	4.863	1.483	0.822–2.678	.191	3.05	1.134	1.01–1.273	.034^*^	
**BPI-pain severity (now)**	Renal dysfunction	1	2.894	0.78–10.74	.112	1	0.736	0.097–5.573	.767	1	7.405	1.205–45.493	.031^*^	
	Hypertension	1	2.182	0.829–5.742	.114	1	0.606	0.142–2.595	.5	1	6.001	1.356–26.564	.018^*^	
	Height	11.4	0.694	0.412–1.168	.169	8.6	1.18	0.542–2.57	.677	8.2	0.455	0.256–0.807	.007^*^	
	Height (Z-score)	1.53	0.616	0.395–0.961	.033^*^	1.478	1.181	0.542–2.574	.676	1.56	0.456	0.257–0.808	.007^*^	
	TUGT	3.385	1.254	1.077–1.46	.004^*^	4.863	2.102	1.084–4.075	.028^*^	3.05	1.193	1.034–1.376	.016^*^	

*(Continued)*

**Table 5:** Continued.

A.														
**Adults**		**All (*N* = 70)**				**Subgroup analysis**								
						**Male (*n =* 24)**				**Female (*n =* 44)**				
**Outcome**	**Exposure**	**Diff (IQR)**	**Odds ratio** **(/IQR)**	**95% confidence interval**	***p***	**Diff (IQR)**	**Odds ratio** **(/IQR)**	**95% confidence interval**	***p***	**Diff (IQR)**	**Odds ratio** **(/IQR)**	**95% confidence interval**	***p***	
**BPI-pain interference**	Hypertension	1	2.296	0.848–6.218	.102	1	0.676	0.156–2.921	.6	1	7.683	1.511–39.07	.014^*^	
	Surgery	1	3.23	1.306–7.988	.011^*^	1	3.098	0.699–13.725	.136	1	3.284	1.042–10.353	.042^*^	
	Height	11.4	0.737	0.448–1.211	.229	8.6	1.214	0.555–2.656	.627	8.2	0.512	0.298–0.88	.015^*^	
	Height (Z-score)	1.53	0.638	0.416–0.98	.04^*^	1.478	1.215	0.555–2.658	.626	1.56	0.513	0.299–0.88	.015^*^	
	TUGT	3.385	1.216	1.029–1.437	.022^*^	4.863	2.496	1.276–4.882	.008^*^	3.05	1.137	1.001–1.292	.049^*^	
**WOMAC Pain**	Hypertension	1	1.856	0.616–5.59	.272	1	0.897	0.216–3.726	.881	1	6.343	1.388–28.995	.017^*^	
	Ectopic ossification and related symptom	1	1.771	0.716–4.377	.216	1	0.479	0.105–2.186	.342	1	3.254	1.079–9.811	.036^*^	
	Hearing impairment	1	2.199	0.299–16.185	.439	1	0.152	0.011–2.09	.159	1	31.743	1.369–735.817	.031^*^	
	Surgery	1	3.641	1.463–9.057	.005^*^	1	6.147	1.226–30.82	.027^*^	1	3.601	1.093–11.863	.035^*^	
	TUGT	3.385	1.252	1.082–1.448	.003^*^	4.863	2.95	1.404–6.196	.004^*^	3.05	1.175	1.034–1.334	.013^*^	
	Grip strength	8	0.617	0.385–0.987	.044^*^	9.5	0.472	0.158–1.406	.178	8	0.304	0.116–0.798	.016^*^	
**WOMAC Stiffness**	Ectopic ossification and related symptoms	1	1.654	0.689–3.969	.26	1	0.634	0.138–2.926	.56	1	3.566	1.042–12.2	.043^*^	
	TUGT	3.385	1.464	1.109–1.933	.007^*^	4.863	3.31	1.413–7.753	.006^*^	3.05	1.367	1.11–1.683	.003^*^	
**WOMAC Physical function**	Hypertension	1	1.731	0.631–4.747	.286	1	0.617	0.148–2.576	.508	1	7.318	1.593–33.625	.011^*^	
	Ectopic ossification and related symptom	1	1.772	0.727–4.319	.208	1	0.413	0.094–1.819	.243	1	4.896	1.439–16.652	.011^*^	
	Hearing impairment	1	2.414	0.383–15.224	.348	1	0.361	0.041–3.197	.36	1	28.991	1.219–689.605	.037^*^	
	Surgery	1	6.314	2.305–17.296	<.001^*^	1	5.164	1.11–24.03	.036^*^	1	7.618	1.866–31.104	.005^*^	
	Height	11.4	0.709	0.402–1.251	.236	8.6	1.288	0.574–2.893	.54	8.2	0.457	0.228–0.914	.027^*^	
	Height (Z-score)	1.53	0.656	0.4–1.075	.094	1.478	1.307	0.588–2.907	.511	1.56	0.457	0.229–0.914	.027^*^	
	TUGT	3.385	1.314	1.092–1.58	.004^*^	4.863	3	1.431–6.291	.004^*^	3.05	1.227	1.008–1.492	.041^*^	
	Grip strength	8	0.664	0.416–1.059	.085	9.5	0.646	0.254–1.642	.358	8	0.3	0.123–0.734	.008^*^	

*(Continued)*

**Table 5:** Continued.

B.													
**School (children)**		**All (*N* = 49)**				**Subgroup analysis**							
						**Male (*n =* 12)**				**Female (*n =* 37)**			
**Outcome**	**Exposure**	**Diff (IQR)**	**Odds ratio** **(/IQR)**	**95% confidence interval**	***p***	**Diff (IQR)**	**Odds ratio** **(/IQR)**	**95% confidence interval**	***p***	**Diff (IQR)**	**Odds ratio** **(/IQR)**	**95% confidence interval**	***p***
**Total days/year of school non-attendance due to symptoms/ complications of XLH**	Ectopic ossification and related symptoms	1	29.751	2.982–296.847	.004^*^	1	19500.433	0–NA	.647	1	4207.293	0–NA	.689
	Height (Z-score)	1.38	1.254	0.428–3.671	.68	0.723	20.336	1.486–278.348	.024^*^	1.52	0.868	0.166–4.553	.867
**Total days/year of school non-attendance due to management of XLH**	Calcification	1	0.375	0.119–1.181	.094	1	5.459	0.219–136.24	.301	1	0.246	0.068–0.889	.032^*^
	Height (Z-score)	1.38	0.854	0.462–1.58	.615	0.723	5.218	1.009–27	.049^*^	1.52	0.68	0.322–1.437	.312
**Work (adults)**		**All (*N* = 24)**				**Subgroup analysis**							
		**Male (*n =* 11)**				**Female (*n =* 13)**							
**Outcome**	**Exposure**	**Diff (IQR)**	**Odds ratio** **(/IQR)**	**95% confidence interval**	***p***	**Diff (IQR)**	**Odds ratio** **(/IQR)**	**95% confidence interval**	***p***	**Diff (IQR)**	**Odds ratio** **(/IQR)**	**95% confidence interval**	***p***
**Total days/year of work non-attendance due to management of XLH**	Renal dysfunction	1	11.651	1.33–102.041	.027^*^	1	17.63	0.716–434.041	.079	1	10	0.317–315.279	.191
	Hypertension	1	6.738	1.24–36.599	.027^*^	1	43.048	1.911–969.873	.018^*^	1	1.678	0.094–29.837	.725
C.													
**School (children)**		**All (*N* = 40)**				**Subgroup analysis**							
						**Male (*n =* 11)**				**Female (*n =* 29)**			
**Outcome**	**Exposure**	**Diff (IQR)**	**Odds ratio** **(/IQR)**	**95% confidence interval**	***p***	**Diff (IQR)**	**Odds ratio** **(/IQR)**	**95% confidence interval**	***p***	**Diff (IQR)**	**Odds ratio** **(/IQR)**	**95% confidence interval**	***p***
**Total days/year of school non-attendance due to symptoms/ complications of XLH**	SF-10: PHS score	13.86	0.067	0.008–0.562	.013^*^	11.81	0.001	0–1.415	.063	10.958	0.469	0.057–3.855	.481
	SF-10: PSS score	8.02	0.162	0.036–0.724	.017^*^	2.675	0.508	0.267–0.963	.038^*^	11.36	0.46	0.011–19.893	.686

*(Continued)*

**Table 5:** Continued.

C.													
**School (children)**		**All (*N* = 40)**				**Subgroup analysis**							
						**Male (*n =* 11)**				**Female (*n =* 29)**			
**Outcome**	**Exposure**	**Diff (IQR)**	**Odds ratio** **(/IQR)**	**95% confidence interval**	***p***	**Diff (IQR)**	**Odds ratio** **(/IQR)**	**95% confidence interval**	***p***	**Diff (IQR)**	**Odds ratio** **(/IQR)**	**95% confidence interval**	***p***
**Total days/year of school non-attendance due to management of XLH**	SF-10: PHS score	13.86	0.476	0.193–1.176	.108	11.81	0.057	0.005–0.61	.018^*^	10.958	0.974	0.358–2.654	.959
**Work (adults)**		**All (*N* = 23)**				**Subgroup analysis**							
						**Male (*n =* 10)**				**Female (*n =* 13)**			
**Outcome**	**Exposure**	**Diff (IQR)**	**Odds ratio** **(/IQR)**	**95% confidence interval**	***p***	**Diff (IQR)**	**Odds ratio** **(/IQR)**	**95% confidence interval**	***p***	**Diff (IQR)**	**Odds ratio** **(/IQR)**	**95% confidence interval**	***p***
**Total days/year of work non-attendance due to symptoms/ complications of XLH**	BPI-pain interference	2.964	7.466	1.335–41.763	.022^*^	2.964	54675.676	0–NA	.84	1.607	2.286	0.823–6.355	.113
	WOMAC Physical function)	12.5	2.53	1.069–5.985	.035^*^	11.397	356.144	0–NA	.751	7.353	2.65	0.703–9.981	.15
**School or work (adults)**		**All (*N* = 31)**				**Subgroup analysis**							
		**Male (*n =* 11)**				**Female (*n =* 20)**							
**Outcome**	**Exposure**	**Diff (IQR)**	**Odds ratio** **(/IQR)**	**95% confidence interval**	***p***	**Diff (IQR)**	**Odds ratio** **(/IQR)**	**95% confidence interval**	***p***	**Diff (IQR)**	**Odds ratio** **(/IQR)**	**95% confidence interval**	***p***
**Total days/year of school/work non-attendance due to symptoms/ complications of XLH**	BPI-pain severity (worst)	5	16.291	1.392–190.693	.026^*^	2	7437.639	0–NA	.742	5	6.273	0.526–74.775	.146
	BPI-pain severity (average)	4	11.41	1.111–117.14	.04^*^	3	854746593.3	0–NA	.753	4	7.254	0.724–72.688	.092
	BPI-pain severity (now)	2	2.717	1.023–7.215	.045^*^	2	1487272.124	0–NA	.777	1	1.365	0.744–2.505	.316
	BPI-pain interference	2.893	4.331	1.197–15.675	.026^*^	2.929	41826.961	0–NA	.833	2.714	2.485	0.609–10.143	.205
	WOMAC Stiffness	25	3.735	1.132–12.33	.031^*^	31.25	86044417.24	0–NA	.792	25	4.122	0.508–33.465	.185
	WOMAC Physical function	14.338	3.044	1.195–7.75	.02^*^	11.765	375.012	0–NA	.737	14.706	3.607	0.556–23.404	.179

IQRs represent the odds ratio corresponding to a change in the explanatory variable of IQR.

Extracted data for items with significant differences. Full data are available in Table S1–S3.

^*^ *p*-value <.05

(A) Significant relationships between SF-10/FPS-R and comorbidity (children) and between BPI/WOMAC and comorbidity (adults), (B) Significant relationships between school status and comorbidity and between work and comorbidity, (C) Significant relationships between school/work status and SF-10, FPS-R, BPI, and WOMAC.

Abbreviations: 6MWT = 6-min walk test; BPI = brief pain inventory; FPS-R = revised faces pain scale; IQR = interquartile range; MAD = mechanical axis deviation; PHS = physical; PSS = psychosocial; R = right; SF-10 = 10-item short-form health survey; TUGT = Timed Up and Go Test; WOMAC = Western Ontario and McMaster Universities osteoarthritis index; XLH = X-linked hypophosphatemic rickets/osteomalacia; NA = not applicable.

The relationships between SF-10/FPS-R and comorbidity in children and between BPI/WOMAC scores and comorbidity in adults are shown in Tables 5A and S1. In children, there was a significant relationship between the SF-10 physical score and both ectopic ossification and related symptoms (*p* =.008), as well as surgery (*p* =.037). Additionally, a significant relationship was found between the SF-10 psychosocial score and hyperparathyroidism (*p* =.029) and surgery (*p* =.021). In male children, there was a significant relationship between SF-10 psychosocial score and the 6MWT (*p* =.041). In female children, significant relationships were found between the SF-10 physical score and bone deformity and related symptoms (*p* =.036), ectopic ossification and related symptoms (*p* =.015), and surgery (*p* =.003), and between SF-10 psychosocial score and surgery (*p* =.0015). In male children, there were significant relationships between FPS-R pain scale and bone deformity and related symptoms (*p* =.032). In adults, there were significant relationships between BPI and hypertension (worst, *p* =.017; least, *p* =.022; average, *p* =.002), height (Z-score) (average, *p* =.048; pain interference, *p* =.04), and TUGT (worst, *p* =.002; least, *p* =.004; average, *p* =.005; now, *p* =.004; pain interference, *p* =.022). In addition, there were significant relationships between WOMAC pain scores and surgery (*p* =.005), TUGT (*p* =.003), and grip strength (*p* =.044). There were notable but non-significant relationships between WOMAC pain scores and ectopic ossification and related symptoms (*p* =.216), and height (Z-score) (*p* =.301). A significant relationship between WOMAC stiffness scores and TUGT was observed (*p* =.007), while the relationship with ectopic ossification and related symptoms was notable but not significant (*p* =.26). There were significant relationships between WOMAC physical function scores and both surgery (*p* <.001) and TUGT (*p* =.004). Although not statistically significant, there appeared to be possible relationships with ectopic ossification and related symptoms (*p* =.286), height (Z-score) (*p* =.094), and grip strength (*p* =.085). In adult males, significant relationships were reported for pain severity and TUGT (worst, now) and grip strength (least); pain interference and TUGT; WOMAC stiffness score and TUGT; and WOMAC physical function score and surgery and TUGT. In adult females, significant relationships were observed between pain severity and hypertension (worst, least, average, pain interference), height/height (Z-score) (worst, average, now, pain interference), and TUGT (worst, least, average, now, pain interference); WOMAC pain score and hypertension, ectopic ossification and related symptoms, hearing impairment, surgery, TUGT, and grip strength; WOMAC stiffness score and ectopic ossification and related symptoms and TUGT; and WOMAC physical function score and hypertension, ectopic ossification and related symptoms, hearing impairment, surgery, height/height (Z-score), TUGT, and grip strength.

The relationships between school status and comorbidity and between work and comorbidity are summarized in Tables 5B and S2. There was a significant relationship between total days of school non-attendance per year due to symptoms/complications of XLH and ectopic ossification and related symptoms with all children (*p* =.004), and with height (Z-score) for male children (*p* =.024). There was also a significant relationship between total days of non-school attendance per year owing to management of XLH and height (Z-score) for male children (*p* =.049), and calcification for female children (*p* =.032). For adults, there was a significant relationship between total days of work non-attendance per year due to management of XLH and renal dysfunction and hypertension (*p* =.027 for both); the relationship with hypertension was also significant for adult males (*p* =.018).

The relationships between school/work status and SF-10, FPS-R, BPI, and WOMAC are summarized in Tables 5C and S3. School non-attendance due to symptoms/complications of XLH had significant relationships with both the SF-10 physical score (*p* =.013) and psychosocial score summaries (*p* =.017) in all children and with the SF-10 psychosocial score summary for male children (*p* =.038). There was also a relationship between school non-attendance due to management of XLH and the SF-10 physical score summary (*p* =.018). For adults, BPI pain interference and WOMAC physical function were significantly related to work non-attendance due to XLH symptoms/complications (*p* =.022 and *p* =.035, respectively). Notably, relationships were more frequently observed between QOL measures and XLH symptoms than between QOL and XLH management.

## Discussion

XLH is a rare genetic disorder^5^ affecting up to one individual per 20 000 to 60 000 live births,^8–10^ and although several observational studies have been conducted to date,^9–19^^,^^32–34^ the limited number of patients in each analysis has hindered widespread analysis of data. In this first analysis of data from the longitudinal, observational SUNFLOWER study, we evaluated baseline information to clarify the physical and mental burden of XLH on affected patients in Japan and South Korea.

In this analysis, most patients received conventional therapy with oral phosphate and/or active vitamin D. Despite this, laboratory test values related to ectopic ossification and related symptoms were observed to be abnormal in study patients: serum phosphate levels and the ratio of tubular maximum reabsorption rate of phosphate to glomerular filtration rate were lower than standard values, while alkaline phosphate (ALP) in children and bone-specific ALP in adults were higher than standard values. The mean height Z-score in our analysis was −2.21 among adults (male, −2.34; female, −2.14), indicating failure to thrive; this suggests a difficulty in normalizing growth rates using conventional therapy. These data are in line with previous data that suggested issues with growth rate and final height cannot be fully resolved by conventional therapy.^35^ However, when looking at height and height Z-score according to age, patients over 40 yr old tended to be shorter than those under 40 yr. This aligns with the period during which the use of current conventional therapy, first studied in the late 1970s, became routine clinical practice in the 1980s^36^^,^^37^ and started to come into routine clinical practice in the 1980s. This may indicate that patients over 40 yr old were undertreated, resulting in a shorter stature. The height of patients under 40 yr old, and particularly for those under 20 yr, may indicate the positive effects of conventional therapy on height. Females 5-12 yr of age tended to be taller than males in this age group, most probably due to differences in the onset of puberty between males and females. Because females generally attain puberty 2 yr earlier than males, the peak of female growth is mostly within the 5-12 age group. In contrast, the peak growth of males spans both the 5-12 and 13-18 age groups. Although there was an overall trend toward lower Z-scores for males than females in relation to age and height Z-scores, there was overlap in the confidence intervals. Similar to this result, previous reports on growth in children with XLH also showed numerically lower height Z-scores for males than for females at ages 1-13 yr, although these differences were not significant.^35^ Interestingly, height and height Z-scores were frequently correlated with pain measures in female adults but not in male adults, potentially indicating a difference between the sexes in the relationship between the frequency of complications and QOL.

The association between *PHEX* mutation and XLH severity is incompletely understood, and the severity of clinical symptoms varied from patient to patient.^38^^,^^39^ This analysis of patients enrolled in the SUNFLOWER study included individuals with relatively mild symptoms (mean pediatric RSS score of 1.62 [male, 2.36; female, 1.10]). This was lower than the RSS scores reported for some clinical trial populations; notably, in the recent randomized, active-controlled, open-label, phase 3 trial of burosumab in XLH, only patients with an RSS score of ≥2.0 were included.^40^ Thus, the SUNFLOWER study population may more accurately reflect real-world clinical practice, as there are thought to be many patients with relatively mild symptoms in daily medical care.

In this analysis, we assessed the various medical complications associated with XLH and their relationship with QOL. Nephrocalcinosis (Grades 1-4, evaluated by renal ultrasound) was observed in 26.0% of children and 41.4% of adults in this analysis. It is known that many patients with XLH develop secondary and tertiary hyperparathyroidism during conventional therapy, and tertiary hyperparathyroidism is a risk factor for renal calcification.^41^ Many patients receive long-term conventional therapy, which may lead to hyperparathyroidism in adults; similarly, renal calcification may be due to iatrogenic adverse reactions to conventional therapy. There were also many complications related to bone deformities (eg, genu varum, genu valgum) in both adults and children in our analysis population. A high incidence of ectopic ossification (eg, osteophytes, enthesopathy) has been reported previously in adults with XLH.^42^ Both of these conditions have been reported as symptoms of XLH that cannot be resolved by conventional therapy.^6^^,^^43^^,^^44^ Notably, some bone-related complications were rarely reported in children (eg, osteophytes, enthesopathy, and spinal stenosis) but were common in adult patients with XLH. Previous studies have reported that the morbidity of these complications increases with age^6^; this is consistent with our findings of higher rates of ectopic ossification and related symptoms among adults compared with children.

Other factors that appeared to affect QOL were pain, motor function, and enthesopathy. The FPS-R and BPI scores indicated that children with XLH had mild pain and adult patients had mild-to-moderate pain in Japan and South Korea. Some patients reported bone pain and arthralgia at baseline, but the degree of pain was relatively mild in many individuals. Despite this, a definite reduction in QOL was noted. There was a significant relationship between both the SF-10 physical score and SF-10 psychosocial score and school non-attendance due to XLH symptoms/complications for all children, and this was more pronounced for males than females. The SF-10 physical score was also significantly correlated with school non-attendance due to XLH management in male children. For adults, pain, physical function, and stiffness were significantly associated with school/work non-attendance due to XLH symptoms/complications. In addition, the mean 6MWT distance of XLH-affected children in this analysis (439.6 m) was shorter than the distance covered by healthy children (655.8-727.6 m),^45^ suggesting that XLH confers issues with motor functioning. In prior clinical studies of children with XLH treated with burosumab,^46^ the mean 6MWT distance at baseline prior to commencing treatment was 483.1 m, which was shorter than that of healthy children, as shown in the present analysis. The adult TUGT score in this study exceeded the diagnostic criterion for locomotor instability of 11 s,^47^ indicating mild motor dysfunction. Grip strength was also lower than the score for the general Japanese population (grip strength data [mean ± SD] in 2022 for healthy males and females aged 34-39 yr, not belonging to a sports club: males, 46.35 ± 7.09 kg; females, 28.36 ± 4.53 kg).^48^ The relationship between grip strength and pain was significant for least pain severity (BPI) in males and WOMAC pain in females. These results are consistent with previous reports of hypophosphatemia affecting muscle strength in patients with XLH.^49^^,^^50^ The relationship between pain and hypertension was significant for worst pain severity (BPI) (*p* =.017) and average pain severity (BPI) (*p* =.002), demonstrating that hypertension was associated with pain in these patients. Both hypertension and renal dysfunction significantly affected work attendance due to treatment management, and the effect of hypertension on work attendance was more pronounced in males than females.

Although many patients did not report absences from work/school due to XLH symptoms, it was notable that some children did require absences of up to 240 d/yr. The mean number of days absent from work/school for XLH treatment was 6.1 d/yr for children and 3.1 d/yr for adults, indicating that XLH may place a burden on patients, either directly or due to the need to act as a caregiver.

Several limitations must be considered when interpreting the data from these analyses. First, since this is a non-randomized observational study, it may be affected by selection bias and confounding factors. The proportional odds logistic regression models did not yield reliable estimates when extremely few subjects and events were in at least one exposure group. Second, this study targeted only patients formally diagnosed with XLH. Patients with mild disease may be unaware of their condition and remain undiagnosed. Thus, the disease course of undiagnosed patients with mild symptoms remains unclear. Third, the effects of surgery and bone fractures were not included in the planned analyses of baseline data. Since these procedures are thought to affect pain and QOL, their impact will be evaluated in future, post-enrollment analyses of the SUNFLOWER population. Finally, it must be noted that the values and conversions implemented for the QOL analyses may not be fully generalizable between the Japanese and South Korean populations in this study, as scoring and index values differ between countries.

In conclusion, this analysis of baseline data from the longitudinal SUNFLOWER study indicates a link between disease and QOL in patients with XLH; we anticipate that these data will be critical in enabling clinicians to understand the daily reality of patients with XLH. To examine the changes over time in each parameter related to patients with XLH and the effects of treatment, multiple future analyses are planned.

## Supplementary Material

Namba_N_et_al_Sunflower_Suppl_tables_0430_ziae118

## Data Availability

Research data, including participant data, the statistical analysis plan, and informed consent forms, are not shared. The study protocol has been previously published.

## Clinical trial registration

NCT03745521; UMIN000031605.
