# Supplementary material for: Impact of X-linked hypophosphatemic rickets/osteomalacia on health and quality of life: baseline data from the SUNFLOWER longitudinal, observational cohort study
Source: JBMR Plus. 2024 Sep 10;8(11):ziae118. doi: 10.1093/jbmrpl/ziae118 (PMC11470975; doi:10.1093/jbmrpl/ziae118)
Supplement: Namba_N_et_al_Sunflower_Suppl_tables_0430_ziae118 [file namba_n_et_al_sunflower_suppl_tables_0430_ziae118.docx]

# Supplementary materials

**Supplementary Table S1.** Relationship between SF-10/FPS-R and comorbidity (children) and between BPI/WOMAC and comorbidity (adults) (full data)

| **Children** | | **All (*N* = 77)** | | | | **Subgroup analysis** | | | | | | | |
| --- | --- | --- | --- | --- | --- | --- | --- | --- | --- | --- | --- | --- | --- |
|  |  |  |  |  |  | **Male (*n* = 25)** | | | | **Female (*n* = 52)** | | | |
| **Outcome** | **Exposure** | **Diff (IQR)** | **Odds ratio (/IQR)** | **95% confidence interval** | ***P*** | **Diff (IQR)** | **Odds ratio (/IQR)** | **95% confidence interval** |  | **Diff (IQR)** | **Odds ratio (/IQR)** | **95% confidence interval** | ***P*** |
| SF-10:  PHS score | Calcification | 1 | 2.26 | 0.892–5.727 | 0.086 | 1 | 2.177 | 0.341–13.908 | 0.411 | 1 | 1.746 | 0.581–5.253 | 0.321 |
|  | Hyperparathyroidism | 1 | 2.392 | 0.665–8.601 | 0.181 | 1 | 1.42 | 0.079–25.572 | 0.812 | 1 | 1.454 | 0.355–5.949 | 0.603 |
|  | Bone deformity and related symptoms | 1 | 0.534 | 0.213–1.336 | 0.18 | 1 | 1.733 | 0.3–10.004 | 0.538 | 1 | 0.312 | 0.105–0.929 | 0.036* |
|  | Abnormal gait | 1 | 1.139 | 0.482–2.692 | 0.766 | 1 | 3.375 | 0.451–25.272 | 0.236 | 1 | 0.609 | 0.165–2.249 | 0.457 |
|  | Bone pain | 1 | 0.833 | 0.254–2.732 | 0.763 | 1 | 1164.574 | 0–NA | 0.77 | 1 | 0.54 | 0.143–2.04 | 0.364 |
|  | Ectopic ossification and related symptom | 1 | 0.27 | 0.102–0.715 | 0.008* | 1 | 0.242 | 0.052–1.116 | 0.069 | 1 | 0.21 | 0.06–0.735 | 0.015* |
|  | Surgery | 1 | 0.331 | 0.117–0.935 | 0.037* | 1 | 1.082 | 0.155–7.574 | 0.937 | 1 | 0.1 | 0.022–0.445 | 0.003* |
|  | Dental problems | 1.344 | 0.526 | 0.155–1.791 | 0.304 | 1 | 0.879 | 0.151–5.101 | 0.885 | 1 | 1.825 | 0.206–16.174 | 0.589 |
|  | Height | 33.375 | 0.849 | 0.375–1.922 | 0.694 | 44.3 | 0.418 | 0.121–1.442 | 0.168 | 41.05 | 1.381 | 0.498–3.828 | 0.535 |
|  | Height (Z-score) | 23.138 | 0.616 | 0.24–1.586 | 0.316 | 0.86 | 0.931 | 0.664–1.307 | 0.681 | 1.647 | 1.828 | 0.675–4.95 | 0.235 |
|  | RSS mean | 96 | 1.331 | 0.665–2.663 | 0.419 | 1.875 | 0.255 | 0.039–1.662 | 0.153 | 1.031 | 0.571 | 0.22–1.479 | 0.248 |
|  | MAD (L) mean | 1.344 | 0.526 | 0.155–1.791 | 0.304 | 30.575 | 1.892 | 0.522–6.863 | 0.332 | 30.75 | 0.425 | 0.128–1.412 | 0.162 |
|  | MAD (R) mean | 33.375 | 0.849 | 0.375–1.922 | 0.694 | 19.625 | 1.216 | 0.552–2.68 | 0.627 | 25.775 | 0.474 | 0.163–1.372 | 0.168 |
|  | 6MWT | 23.138 | 0.616 | 0.24–1.586 | 0.316 | 104.5 | 2.658 | 0.737–9.581 | 0.135 | 98.25 | 0.92 | 0.434–1.951 | 0.829 |
| SF-10:  PSS score | Calcification | 1 | 0.902 | 0.368–2.212 | 0.821 | 1 | 1 | 0.225–4.442 | 1 | 1 | 0.726 | 0.242–2.177 | 0.568 |
|  | Hyperparathyroidism | 1 | 5.325 | 1.187–23.891 | 0.029* | 1 | 1.566 | 0.085–28.982 | 0.763 | 1 | 8.537 | 0.643–113.417 | 0.104 |
|  | Bone deformity and related symptoms | 1 | 0.915 | 0.371–2.259 | 0.847 | 1 | 1.231 | 0.185–8.191 | 0.83 | 1 | 0.983 | 0.341–2.836 | 0.975 |
|  | Abnormal gait | 1 | 1.298 | 0.569–2.962 | 0.535 | 1 | 1.794 | 0.298–10.799 | 0.524 | 1 | 1.16 | 0.412–3.264 | 0.778 |
|  | Bone pain | 1 | 0.612 | 0.173–2.165 | 0.446 | 1 | 2526.867 | 0–NA | 0.787 | 1 | 0.39 | 0.105–1.448 | 0.159 |
|  | Ectopic ossification and related symptom | 1 | 0.397 | 0.143–1.103 | 0.076 | 1 | 0.286 | 0.065–1.271 | 0.1 | 1 | 0.361 | 0.104–1.253 | 0.109 |
|  | Surgery | 1 | 0.294 | 0.104–0.832 | 0.021* | 1 | 0.601 | 0.089–4.075 | 0.603 | 1 | 0.158 | 0.036–0.695 | 0.015* |
|  | Dental problems | 1 | 0.45 | 0.093–2.175 | 0.32 | 1 | 0.78 | 0.095–6.385 | 0.817 | 1 | 0.779 | 0.084–7.23 | 0.826 |
|  | Height | 43.6 | 1.189 | 0.309–4.575 | 0.801 | 44.3 | 0.443 | 0.042–4.622 | 0.496 | 41.05 | 0.711 | 0.326–1.551 | 0.391 |
|  | Height (Z-score) | 1.22 | 1.187 | 0.791–1.782 | 0.407 | 0.86 | 0.952 | 0.655–1.385 | 0.798 | 1.647 | 2.22 | 0.929–5.305 | 0.073 |
|  | RSS mean | 1.344 | 0.689 | 0.268–1.775 | 0.441 | 1.875 | 0.343 | 0.068–1.738 | 0.196 | 1.031 | 0.701 | 0.194–2.535 | 0.588 |
|  | MAD (L) mean | 33.375 | 1.085 | 0.436–2.696 | 0.861 | 30.575 | 1.289 | 0.449–3.698 | 0.637 | 30.75 | 0.989 | 0.319–3.072 | 0.985 |
|  | MAD (R) mean | 23.138 | 0.782 | 0.371–1.649 | 0.518 | 19.625 | 1.1 | 0.408–2.964 | 0.851 | 25.775 | 0.92 | 0.342–2.472 | 0.868 |
|  | 6MWT | 96 | 1.078 | 0.401–2.894 | 0.882 | 104.5 | 3.968 | 1.055–14.919 | 0.041* | 98.25 | 0.792 | 0.402–1.562 | 0.501 |
| FPS-R | Calcification | 1 | 0.876 | 0.272–2.825 | 0.825 | 1 | 2 | 0.256–15.623 | 0.509 | 1 | 0.6 | 0.14–2.563 | 0.49 |
|  | Hyperparathyroidism | 1 | 0.52 | 0.058–4.626 | 0.557 | 1 | 0.004 | 0–NA | 0.853 | 1 | 0.567 | 0.06–5.353 | 0.62 |
|  | Bone deformity and related symptoms | 1 | 0.368 | 0.118–1.145 | 0.084 | 1 | 0.061 | 0.005–0.786 | 0.032* | 1 | 0.826 | 0.224–3.047 | 0.773 |
|  | Abnormal gait | 1 | 0.602 | 0.189–1.914 | 0.39 | 1 | 0.001 | 0–NA | 0.748 | 1 | 0.924 | 0.224–3.81 | 0.913 |
|  | Bone pain | 1 | 1.972 | 0.399–9.749 | 0.405 | 1 | 0.004 | 0–NA | 0.853 | 1 | 2.625 | 0.502–13.719 | 0.253 |
|  | Ectopic ossification and related symptom | 1 | 1.769 | 0.556–5.629 | 0.334 | 1 | 1.238 | 0.166–9.253 | 0.835 | 1 | 2.165 | 0.452–10.379 | 0.334 |
|  | Surgery | 1 | 2.447 | 0.547–10.955 | 0.242 | 1 | 1.447 | 0.187–11.169 | 0.723 | 1 | 4.25 | 0.988–18.289 | 0.052 |
|  | Dental problems | 1 | 0.635 | 0.069–5.821 | 0.688 | 1 | 1.417 | 0.115–17.46 | 0.786 | 1 | 0.001 | 0–NA | 0.834 |
|  | Height | 43.6 | 0.744 | 0.143–3.871 | 0.725 | 44.3 | 0.624 | 0.119–3.281 | 0.578 | 41.05 | 1.187 | 0.193–7.3 | 0.853 |
|  | Height (Z-score) | 1.22 | 0.699 | 0.418–1.168 | 0.172 | 0.86 | 0.765 | 0.485–1.206 | 0.249 | 1.647 | 0.568 | 0.216–1.492 | 0.251 |
|  | RSS mean | 1.344 | 0.929 | 0.403–2.141 | 0.863 | 1.875 | 1.418 | 0.188–10.702 | 0.735 | 1.031 | 1.188 | 0.332–4.256 | 0.791 |
|  | MAD (L) mean | 33.375 | 0.776 | 0.263–2.284 | 0.645 | 30.575 | 0.206 | 0.017–2.487 | 0.214 | 30.75 | 1.536 | 0.398–5.926 | 0.533 |
|  | MAD (R) mean | 23.138 | 1.196 | 0.473–3.023 | 0.705 | 19.625 | 0.404 | 0.052–3.158 | 0.387 | 25.775 | 2.167 | 0.635–7.391 | 0.217 |
|  | 6MWT | 96 | 0.513 | 0.162–1.627 | 0.257 | 104.5 | 0.342 | 0.058–2.032 | 0.238 | 98.25 | 0.508 | 0.145–1.785 | 0.291 |
| **Adults** | | **All (*N* = 70)** | | | | **Subgroup analysis** | | | | | | | |
|  |  |  |  |  |  | **Male (*n* = 24)** | | | | **Female (*n* = 46)** | | | |
| **Outcome** | **Exposure** | **Diff (IQR)** | **Odds ratio (/IQR)** | **95% confidence interval** | ***P*** | **Diff (IQR)** | **Odds ratio (/IQR)** | **95% confidence interval** | ***P*** | **Diff (IQR)** | **Odds ratio (/IQR)** | **95% confidence interval** | ***P*** |
| BPI-pain severity (worst) | Renal dysfunction | 1 | 2.007 | 0.547–7.358 | 0.293 | 1 | 0.358 | 0.042–3.071 | 0.349 | 1 | 5.243 | 0.967–28.435 | 0.055 |
|  | Hypertension | 1 | 3.267 | 1.231–8.672 | 0.017* | 1 | 0.897 | 0.218–3.691 | 0.881 | 1 | 11.485 | 2.478–53.232 | 0.002* |
|  | Calcification | 1 | 1.357 | 0.585–3.149 | 0.477 | 1 | 0.58 | 0.14–2.411 | 0.454 | 1 | 2.036 | 0.699–5.932 | 0.193 |
|  | Hyperparathyroidism | 1 | 1.552 | 0.656–3.669 | 0.317 | 1 | 0.838 | 0.201–3.492 | 0.808 | 1 | 2.118 | 0.677–6.629 | 0.197 |
|  | Bone deformity and related symptoms | 1 | 0.934 | 0.396–2.202 | 0.876 | 1 | 1.591 | 0.367–6.889 | 0.535 | 1 | 0.79 | 0.267–2.341 | 0.671 |
|  | Abnormal gait | 1 | 1.545 | 0.538–4.432 | 0.419 | 1 | 1.028 | 0.208–5.084 | 0.973 | 1 | 1.911 | 0.456–8.02 | 0.376 |
|  | Bone pain | 1 | 1.512 | 0.646–3.538 | 0.341 | 1 | 1 | 0.244–4.093 | 1 | 1 | 1.695 | 0.572–5.028 | 0.341 |
|  | Ectopic ossification and related symptom | 1 | 1.08 | 0.464–2.51 | 0.859 | 1 | 0.416 | 0.093–1.86 | 0.251 | 1 | 1.518 | 0.53–4.353 | 0.437 |
|  | Hearing impairment | 1 | 1.711 | 0.282–10.37 | 0.559 | 1 | 1.3 | 0.145–11.679 | 0.815 | 1 | 2.751 | 0.09–84.422 | 0.562 |
|  | Surgery | 1 | 2.204 | 0.937–5.185 | 0.07 | 1 | 3.088 | 0.707–13.49 | 0.134 | 1 | 1.55 | 0.478–5.023 | 0.465 |
|  | Dental problems | 1 | 1.29 | 0.455–3.654 | 0.632 | 1 | 0.854 | 0.18–4.059 | 0.843 | 1 | 1.579 | 0.367–6.8 | 0.54 |
|  | Height | 11.4 | 0.779 | 0.472–1.285 | 0.328 | 8.6 | 1.331 | 0.603–2.939 | 0.48 | 8.2 | 0.562 | 0.321–0.984 | 0.044* |
|  | Height (Z-score) | 1.53 | 0.688 | 0.446–1.062 | 0.091 | 1.478 | 1.332 | 0.603–2.944 | 0.478 | 1.56 | 0.563 | 0.322–0.984 | 0.044* |
|  | TUGT | 3.385 | 1.237 | 1.082–1.416 | 0.002* | 4.863 | 2.276 | 1.182–4.382 | 0.014* | 3.05 | 1.19 | 1.037–1.366 | 0.013* |
|  | Grip strength | 8 | 0.827 | 0.531–1.288 | 0.4 | 9.5 | 0.601 | 0.22–1.643 | 0.321 | 8 | 0.501 | 0.2–1.252 | 0.139 |
| BPI-pain severity (least) | Renal dysfunction | 1 | 3.078 | 0.797–11.882 | 0.103 | 1 | 0.957 | 0.127–7.198 | 0.966 | 1 | 6.885 | 1.073–44.154 | 0.042* |
|  | Hypertension | 1 | 3.209 | 1.179–8.732 | 0.022* | 1 | 1.152 | 0.264–5.034 | 0.851 | 1 | 5.793 | 1.278–26.271 | 0.023* |
|  | Calcification | 1 | 2.911 | 1.101–7.698 | 0.031* | 1 | 1.679 | 0.382–7.367 | 0.493 | 1 | 4.601 | 1.178–17.97 | 0.028* |
|  | Hyperparathyroidism | 1 | 2.241 | 0.858–5.858 | 0.1 | 1 | 1.267 | 0.289–5.566 | 0.754 | 1 | 2.617 | 0.705–9.722 | 0.151 |
|  | Bone deformity and related symptoms | 1 | 0.921 | 0.344–2.465 | 0.869 | 1 | 1.163 | 0.254–5.324 | 0.846 | 1 | 0.753 | 0.194–2.922 | 0.682 |
|  | Abnormal gait | 1 | 2.093 | 0.597–7.339 | 0.249 | 1 | 1.068 | 0.188–6.055 | 0.941 | 1 | 2.754 | 0.519–14.616 | 0.234 |
|  | Bone pain | 1 | 0.816 | 0.302–2.204 | 0.688 | 1 | 0.888 | 0.203–3.894 | 0.875 | 1 | 0.683 | 0.175–2.656 | 0.582 |
|  | Ectopic ossification and related symptom | 1 | 1.891 | 0.714–5.007 | 0.2 | 1 | 0.605 | 0.127–2.88 | 0.528 | 1 | 2.596 | 0.666–10.113 | 0.169 |
|  | Hearing impairment | 1 | 4.257 | 0.753–24.071 | 0.101 | 1 | 3 | 0.33–27.234 | 0.329 | 1 | 5.332 | 0.284–100.033 | 0.263 |
|  | Surgery | 1 | 1.908 | 0.711–5.116 | 0.2 | 1 | 1.369 | 0.308–6.094 | 0.68 | 1 | 2.551 | 0.704–9.248 | 0.154 |
|  | Dental problems | 1 | 1.736 | 0.575–5.238 | 0.328 | 1 | 0.947 | 0.194–4.626 | 0.946 | 1 | 2.204 | 0.444–10.932 | 0.334 |
|  | Height | 11.4 | 0.903 | 0.509–1.6 | 0.726 | 8.6 | 0.971 | 0.443–2.125 | 0.941 | 8.2 | 0.564 | 0.313–1.016 | 0.057 |
|  | Height (Z–score) | 1.53 | 0.654 | 0.41–1.044 | 0.075 | 1.478 | 0.97 | 0.443–2.125 | 0.94 | 1.56 | 0.563 | 0.313–1.013 | 0.055 |
|  | TUGT | 3.385 | 1.373 | 1.104–1.708 | 0.004* | 4.863 | 1.378 | 0.735–2.584 | 0.317 | 3.05 | 1.288 | 1.051–1.578 | 0.015 |
|  | Grip strength | 8 | 0.832 | 0.519–1.334 | 0.446 | 9.5 | 0.268 | 0.081–0.881 | 0.03* | 8 | 0.653 | 0.262–1.627 | 0.36 |
| BPI-pain severity (average) | Renal dysfunction | 1 | 4.067 | 1.05–15.751 | 0.042* | 1 | 1.474 | 0.158–13.718 | 0.733 | 1 | 7.644 | 1.321–44.221 | 0.023* |
|  | Hypertension | 1 | 4.787 | 1.782–12.861 | 0.002* | 1 | 1.167 | 0.28–4.86 | 0.832 | 1 | 15.601 | 3.392–71.764 | < 0.001* |
|  | Calcification | 1 | 1.576 | 0.673–3.689 | 0.294 | 1 | 0.936 | 0.226–3.887 | 0.928 | 1 | 1.986 | 0.667–5.91 | 0.218 |
|  | Hyperparathyroidism | 1 | 1.861 | 0.781–4.435 | 0.161 | 1 | 1.276 | 0.305–5.333 | 0.739 | 1 | 2.113 | 0.683–6.536 | 0.194 |
|  | Bone deformity and related symptoms | 1 | 0.754 | 0.315–1.807 | 0.526 | 1 | 1.358 | 0.311–5.936 | 0.684 | 1 | 0.523 | 0.164–1.666 | 0.273 |
|  | Abnormal gait | 1 | 1.665 | 0.574–4.825 | 0.348 | 1 | 1.16 | 0.222–6.072 | 0.861 | 1 | 2.067 | 0.473–9.033 | 0.334 |
|  | Bone pain | 1 | 1.446 | 0.577–3.623 | 0.432 | 1 | 1.485 | 0.357–6.182 | 0.587 | 1 | 1.639 | 0.553–4.855 | 0.373 |
|  | Ectopic ossification and related symptom | 1 | 1.462 | 0.626–3.411 | 0.38 | 1 | 0.602 | 0.139–2.604 | 0.497 | 1 | 2.029 | 0.698–5.896 | 0.194 |
|  | Hearing impairment | 1 | 3.769 | 0.685–20.737 | 0.127 | 1 | 1.522 | 0.17–13.637 | 0.707 | 1 | 10.267 | 0.626–168.454 | 0.103 |
|  | Surgery | 1 | 2.242 | 0.948–5.301 | 0.066 | 1 | 2.317 | 0.546–9.834 | 0.254 | 1 | 2.106 | 0.696–6.37 | 0.187 |
|  | Dental problems | 1 | 1.345 | 0.457–3.961 | 0.591 | 1 | 0.831 | 0.174–3.967 | 0.816 | 1 | 1.435 | 0.302–6.817 | 0.65 |
|  | Height | 11.4 | 0.746 | 0.455–1.224 | 0.246 | 8.6 | 0.933 | 0.42–2.072 | 0.864 | 8.2 | 0.521 | 0.302–0.9 | 0.019* |
|  | Height (Z-score) | 1.53 | 0.64 | 0.411–0.997 | 0.048* | 1.478 | 0.934 | 0.42–2.076 | 0.867 | 1.56 | 0.522 | 0.303–0.901 | 0.019* |
|  | TUGT | 3.385 | 1.218 | 1.06–1.4 | 0.005* | 4.863 | 1.483 | 0.822–2.678 | 0.191 | 3.05 | 1.134 | 1.01–1.273 | 0.034* |
|  | Grip strength | 8 | 0.87 | 0.566–1.338 | 0.526 | 9.5 | 0.832 | 0.319–2.17 | 0.707 | 8 | 0.442 | 0.186–1.049 | 0.064 |
| BPI-pain severity (now) | Renal dysfunction | 1 | 2.894 | 0.78-10.74 | 0.112 | 1 | 0.736 | 0.097–5.573 | 0.767 | 1 | 7.405 | 1.205-45.493 | 0.031* |
|  | Hypertension | 1 | 2.182 | 0.829-5.742 | 0.114 | 1 | 0.606 | 0.142–2.595 | 0.5 | 1 | 6.001 | 1.356-26.564 | 0.018* |
|  | Calcification | 1 | 1.734 | 0.69-4.359 | 0.242 | 1 | 1.502 | 0.353–6.382 | 0.582 | 1 | 1.997 | 0.64-6.229 | 0.234 |
|  | Hyperparathyroidism | 1 | 1.626 | 0.661-3.997 | 0.29 | 1 | 1.254 | 0.295–5.333 | 0.759 | 1 | 1.625 | 0.491-5.373 | 0.426 |
|  | Bone deformity and related symptoms | 1 | 0.801 | 0.315-2.038 | 0.642 | 1 | 1.478 | 0.329–6.635 | 0.61 | 1 | 0.546 | 0.157-1.902 | 0.342 |
|  | Abnormal gait | 1 | 2.072 | 0.675-6.357 | 0.203 | 1 | 1.648 | 0.303–8.951 | 0.563 | 1 | 2.178 | 0.465-10.206 | 0.323 |
|  | Bone pain | 1 | 1.576 | 0.643–3.86 | 0.32 | 1 | 1.589 | 0.367–6.871 | 0.535 | 1 | 1.45 | 0.458–4.59 | 0.528 |
|  | Ectopic ossification and related symptom | 1 | 1.602 | 0.655–3.916 | 0.302 | 1 | 1.08 | 0.231–5.052 | 0.922 | 1 | 1.791 | 0.575–5.575 | 0.315 |
|  | Hearing impairment | 1 | 3.023 | 0.488–18.719 | 0.234 | 1 | 2.312 | 0.265–20.208 | 0.449 | 1 | 8.878 | 0.146–540.384 | 0.298 |
|  | Surgery | 1 | 2.23 | 0.905–5.493 | 0.081 | 1 | 2.147 | 0.492–9.371 | 0.31 | 1 | 2.12 | 0.667–6.742 | 0.203 |
|  | Dental problems | 1 | 1.317 | 0.452–3.841 | 0.614 | 1 | 1.026 | 0.221–4.764 | 0.974 | 1 | 1.383 | 0.27–7.083 | 0.697 |
|  | Height | 11.4 | 0.694 | 0.412–1.168 | 0.169 | 8.6 | 1.18 | 0.542–2.57 | 0.677 | 8.2 | 0.455 | 0.256–0.807 | 0.007* |
|  | Height (Z–score) | 1.53 | 0.616 | 0.395–0.961 | 0.033* | 1.478 | 1.181 | 0.542–2.574 | 0.676 | 1.56 | 0.456 | 0.257–0.808 | 0.007* |
|  | TUGT | 3.385 | 1.254 | 1.077–1.46 | 0.004* | 4.863 | 2.102 | 1.084–4.075 | 0.028* | 3.05 | 1.193 | 1.034–1.376 | 0.016* |
|  | Grip strength | 8 | 0.821 | 0.535–1.261 | 0.369 | 9.5 | 0.469 | 0.174–1.267 | 0.135 | 8 | 0.521 | 0.17–1.594 | 0.253 |
| BPI-pain interference | Renal dysfunction | 1 | 1.453 | 0.389–5.42 | 0.578 | 1 | 0.241 | 0.02–2.893 | 0.262 | 1 | 3.837 | 0.691–21.317 | 0.124 |
|  | Hypertension | 1 | 2.296 | 0.848–6.218 | 0.102 | 1 | 0.676 | 0.156–2.921 | 0.6 | 1 | 7.683 | 1.511–39.07 | 0.014* |
|  | Calcification | 1 | 1.226 | 0.525–2.866 | 0.638 | 1 | 0.686 | 0.163–2.878 | 0.606 | 1 | 1.76 | 0.591–5.239 | 0.31 |
|  | Hyperparathyroidism | 1 | 1.53 | 0.633–3.699 | 0.345 | 1 | 0.571 | 0.134–2.424 | 0.447 | 1 | 2.216 | 0.697–7.044 | 0.177 |
|  | Bone deformity and related symptoms | 1 | 1.052 | 0.442–2.501 | 0.909 | 1 | 1.867 | 0.431–8.091 | 0.404 | 1 | 0.832 | 0.277–2.498 | 0.743 |
|  | Abnormal gait | 1 | 1.895 | 0.603–5.953 | 0.274 | 1 | 1.157 | 0.225–5.961 | 0.861 | 1 | 2.666 | 0.613–11.599 | 0.191 |
|  | Bone pain | 1 | 1.603 | 0.675–3.806 | 0.285 | 1 | 1.422 | 0.341–5.919 | 0.629 | 1 | 1.597 | 0.531–4.804 | 0.405 |
|  | Ectopic ossification and related symptom | 1 | 1.467 | 0.599–3.595 | 0.402 | 1 | 0.322 | 0.067–1.557 | 0.159 | 1 | 3.095 | 0.931–10.292 | 0.065 |
|  | Hearing impairment | 1 | 1.507 | 0.259–8.756 | 0.648 | 1 | 0.53 | 0.038–7.32 | 0.635 | 1 | 6.411 | 0.353–116.289 | 0.209 |
|  | Surgery | 1 | 3.23 | 1.306–7.988 | 0.011* | 1 | 3.098 | 0.699–13.725 | 0.136 | 1 | 3.284 | 1.042–10.353 | 0.042* |
|  | Dental problems | 1 | 1.183 | 0.411–3.408 | 0.756 | 1 | 0.544 | 0.109–2.725 | 0.459 | 1 | 1.913 | 0.432–8.472 | 0.393 |
|  | Height | 11.4 | 0.737 | 0.448–1.211 | 0.229 | 8.6 | 1.214 | 0.555–2.656 | 0.627 | 8.2 | 0.512 | 0.298–0.88 | 0.015* |
|  | Height (Z-score) | 1.53 | 0.638 | 0.416–0.98 | 0.04* | 1.478 | 1.215 | 0.555–2.658 | 0.626 | 1.56 | 0.513 | 0.299–0.88 | 0.015* |
|  | TUGT | 3.385 | 1.216 | 1.029–1.437 | 0.022* | 4.863 | 2.496 | 1.276–4.882 | 0.008* | 3.05 | 1.137 | 1.001–1.292 | 0.049* |
|  | Grip strength | 8 | 0.707 | 0.448–1.114 | 0.135 | 9.5 | 0.391 | 0.122–1.254 | 0.114 | 8 | 0.437 | 0.181–1.055 | 0.066 |
| WOMAC Pain | Renal dysfunction | 1 | 0.703 | 0.176–2.805 | 0.618 | 1 | 0.445 | 0.068–2.898 | 0.397 | 1 | 1.128 | 0.176–7.244 | 0.899 |
|  | Hypertension | 1 | 1.856 | 0.616–5.59 | 0.272 | 1 | 0.897 | 0.216–3.726 | 0.881 | 1 | 6.343 | 1.388–28.995 | 0.017* |
|  | Calcification | 1 | 0.862 | 0.374–1.99 | 0.729 | 1 | 0.936 | 0.226–3.878 | 0.928 | 1 | 1.006 | 0.357–2.832 | 0.991 |
|  | Hyperparathyroidism | 1 | 1.029 | 0.431–2.457 | 0.949 | 1 | 0.71 | 0.169–2.981 | 0.64 | 1 | 1.322 | 0.43–4.061 | 0.626 |
|  | Bone deformity and related symptoms | 1 | 1.089 | 0.461–2.57 | 0.846 | 1 | 0.824 | 0.186–3.648 | 0.799 | 1 | 1.039 | 0.352–3.067 | 0.944 |
|  | Abnormal gait | 1 | 1.329 | 0.462–3.822 | 0.598 | 1 | 1.526 | 0.314–7.425 | 0.6 | 1 | 1.576 | 0.386–6.436 | 0.526 |
|  | Bone pain | 1 | 1.598 | 0.679–3.763 | 0.283 | 1 | 1.274 | 0.309–5.259 | 0.738 | 1 | 1.574 | 0.52–4.762 | 0.422 |
|  | Ectopic ossification and related symptom | 1 | 1.771 | 0.716–4.377 | 0.216 | 1 | 0.479 | 0.105–2.186 | 0.342 | 1 | 3.254 | 1.079–9.811 | 0.036* |
|  | Hearing impairment | 1 | 2.199 | 0.299–16.185 | 0.439 | 1 | 0.152 | 0.011–2.09 | 0.159 | 1 | 31.743 | 1.369–735.817 | 0.031* |
|  | Surgery | 1 | 3.641 | 1.463–9.057 | 0.005* | 1 | 6.147 | 1.226–30.82 | 0.027* | 1 | 3.601 | 1.093–11.863 | 0.035* |
|  | Dental problems | 1 | 0.841 | 0.284–2.486 | 0.754 | 1 | 0.27 | 0.051–1.432 | 0.124 | 1 | 1.406 | 0.365–5.418 | 0.62 |
|  | Height | 11.4 | 0.761 | 0.453–1.277 | 0.301 | 8.6 | 1.201 | 0.533–2.709 | 0.659 | 8.2 | 0.62 | 0.354–1.087 | 0.095 |
|  | Height (Z-score) | 1.53 | 0.756 | 0.46–1.244 | 0.271 | 1.478 | 1.201 | 0.532–2.71 | 0.659 | 1.56 | 0.621 | 0.355–1.087 | 0.095 |
|  | TUGT | 3.385 | 1.252 | 1.082–1.448 | 0.003* | 4.863 | 2.95 | 1.404–6.196 | 0.004* | 3.05 | 1.175 | 1.034–1.334 | 0.013* |
|  | Grip strength | 8 | 0.617 | 0.385–0.987 | 0.044* | 9.5 | 0.472 | 0.158–1.406 | 0.178 | 8 | 0.304 | 0.116–0.798 | 0.016* |
| WOMAC  Stiffness | Renal dysfunction | 1 | 0.858 | 0.186–3.963 | 0.844 | 1 | 0.505 | 0.053–4.815 | 0.552 | 1 | 1.433 | 0.249–8.23 | 0.687 |
|  | Hypertension | 1 | 1.417 | 0.496–4.047 | 0.515 | 1 | 0.798 | 0.188–3.38 | 0.76 | 1 | 1.914 | 0.484–7.574 | 0.355 |
|  | Calcification | 1 | 1.356 | 0.569–3.23 | 0.491 | 1 | 0.799 | 0.189–3.37 | 0.76 | 1 | 2.632 | 0.809–8.557 | 0.108 |
|  | Hyperparathyroidism | 1 | 1.421 | 0.564–3.577 | 0.456 | 1 | 0.913 | 0.215–3.872 | 0.902 | 1 | 1.571 | 0.488–5.056 | 0.449 |
|  | Bone deformity and related symptoms | 1 | 1.708 | 0.706–4.129 | 0.235 | 1 | 1.552 | 0.346–6.971 | 0.566 | 1 | 1.597 | 0.511–4.987 | 0.42 |
|  | Abnormal gait | 1 | 1.604 | 0.519–4.953 | 0.412 | 1 | 1.348 | 0.263–6.92 | 0.72 | 1 | 1.773 | 0.424–7.417 | 0.433 |
|  | Bone pain | 1 | 1.226 | 0.507–2.969 | 0.651 | 1 | 1.314 | 0.308–5.599 | 0.712 | 1 | 0.93 | 0.3–2.883 | 0.9 |
|  | Ectopic ossification and related symptom | 1 | 1.654 | 0.689–3.969 | 0.26 | 1 | 0.634 | 0.138–2.926 | 0.56 | 1 | 3.566 | 1.042–12.2 | 0.043* |
|  | Hearing impairment | 1 | 1.438 | 0.279–7.409 | 0.664 | 1 | 0.499 | 0.037–6.799 | 0.602 | 1 | 3.07 | 0.349–27.035 | 0.312 |
|  | Surgery | 1 | 2.122 | 0.824–5.463 | 0.119 | 1 | 1.859 | 0.432–7.991 | 0.405 | 1 | 2.829 | 0.825–9.704 | 0.098 |
|  | Dental problems | 1 | 0.718 | 0.236–2.183 | 0.56 | 1 | 0.246 | 0.04–1.495 | 0.128 | 1 | 1.45 | 0.372–5.654 | 0.592 |
|  | Height | 11.4 | 1.027 | 0.589–1.79 | 0.925 | 8.6 | 1.212 | 0.542–2.707 | 0.639 | 8.2 | 0.704 | 0.354–1.397 | 0.315 |
|  | Height (Z-score) | 1.53 | 0.899 | 0.565–1.429 | 0.651 | 1.478 | 1.211 | 0.542–2.706 | 0.641 | 1.56 | 0.64 | 0.303–1.351 | 0.242 |
|  | TUGT | 3.385 | 1.464 | 1.109–1.933 | 0.007* | 4.863 | 3.31 | 1.413–7.753 | 0.006* | 3.05 | 1.367 | 1.11–1.683 | 0.003* |
|  | Grip strength | 8 | 0.774 | 0.5–1.196 | 0.248 | 9.5 | 0.33 | 0.108–1.003 | 0.051 | 8 | 0.625 | 0.289–1.352 | 0.233 |
| WOMAC Physical function | Renal dysfunction | 1 | 0.628 | 0.16–2.465 | 0.505 | 1 | 0.133 | 0.011–1.61 | 0.113 | 1 | 1.363 | 0.232–7.999 | 0.732 |
|  | Hypertension | 1 | 1.731 | 0.631–4.747 | 0.286 | 1 | 0.617 | 0.148–2.576 | 0.508 | 1 | 7.318 | 1.593–33.625 | 0.011* |
|  | Calcification | 1 | 0.818 | 0.349–1.915 | 0.643 | 1 | 0.566 | 0.13–2.456 | 0.447 | 1 | 1.416 | 0.484–4.143 | 0.525 |
|  | Hyperparathyroidism | 1 | 0.818 | 0.339–1.974 | 0.655 | 1 | 0.388 | 0.089–1.694 | 0.208 | 1 | 1.3 | 0.425–3.97 | 0.645 |
|  | Bone deformity and related symptoms | 1 | 1.26 | 0.534–2.974 | 0.598 | 1 | 0.709 | 0.165–3.047 | 0.644 | 1 | 1.285 | 0.428–3.854 | 0.655 |
|  | Abnormal gait | 1 | 1.906 | 0.664–5.472 | 0.231 | 1 | 1.865 | 0.408–8.534 | 0.422 | 1 | 2.012 | 0.472–8.574 | 0.344 |
|  | Bone pain | 1 | 1.284 | 0.49–3.366 | 0.611 | 1 | 0.76 | 0.178–3.247 | 0.711 | 1 | 1.503 | 0.52–4.345 | 0.452 |
|  | Ectopic ossification and related symptom | 1 | 1.772 | 0.727–4.319 | 0.208 | 1 | 0.413 | 0.094–1.819 | 0.243 | 1 | 4.896 | 1.439–16.652 | 0.011* |
|  | Hearing impairment | 1 | 2.414 | 0.383–15.224 | 0.348 | 1 | 0.361 | 0.041–3.197 | 0.36 | 1 | 28.991 | 1.219–689.605 | 0.037* |
|  | Surgery | 1 | 6.314 | 2.305–17.296 | < 0.001* | 1 | 5.164 | 1.11–24.03 | 0.036* | 1 | 7.618 | 1.866–31.104 | 0.005* |
|  | Dental problems | 1 | 0.879 | 0.316–2.451 | 0.806 | 1 | 0.268 | 0.045–1.588 | 0.147 | 1 | 1.97 | 0.521–7.447 | 0.318 |
|  | Height | 11.4 | 0.709 | 0.402–1.251 | 0.236 | 8.6 | 1.288 | 0.574–2.893 | 0.54 | 8.2 | 0.457 | 0.228–0.914 | 0.027* |
|  | Height (Z-score) | 1.53 | 0.656 | 0.4–1.075 | 0.094 | 1.478 | 1.307 | 0.588–2.907 | 0.511 | 1.56 | 0.457 | 0.229–0.914 | 0.027* |
|  | TUGT | 3.385 | 1.314 | 1.092–1.58 | 0.004* | 4.863 | 3 | 1.431–6.291 | 0.004* | 3.05 | 1.227 | 1.008–1.492 | 0.041* |
|  | Grip strength | 8 | 0.664 | 0.416–1.059 | 0.085 | 9.5 | 0.646 | 0.254–1.642 | 0.358 | 8 | 0.3 | 0.123–0.734 | 0.008* |

/IQRs represent the odds ratio corresponding to a change in the explanatory variable of IQR.

**P*-value <0.05

6MWT = 6-minute walk test; BPI = brief pain inventory; FPS-R = revised faces pain scale; IQR = interquartile range; L = left; MAD = mechanical axis deviation; PHS = physical; PSS = psychosocial; R = right; RSS = Rickets severity score; SF-10 = 10-item short-form health survey; TUGT = Timed Up and Go Test; WOMAC = Western Ontario and McMaster Universities osteoarthritis index.

**Supplementary Table S2.** Relationships between school status and comorbidity and between work and comorbidity (full data)

| **School (children)** | | **All (*N* = 49)** | | | | **Subgroup analysis** | | | | | | | |
| --- | --- | --- | --- | --- | --- | --- | --- | --- | --- | --- | --- | --- | --- |
|  |  |  |  |  |  | **Male (*n* = 12)** | | | | **Female (*n* = 37)** | | | |
| **Outcome** | **Exposure** | **Diff (IQR)** | **Odds ratio (/IQR)** | **95% confidence interval** | ***P*** | **Diff (IQR)** | **Odds ratio (/IQR)** | **95% confidence interval** | ***P*** | **Diff (IQR)** | **Odds ratio (/IQR)** | **95% confidence interval** | ***P*** |
| Total days/year of school non-attendance due to symptoms/ complications of XLH | Calcification | 1 | 2.775 | 0.491–15.672 | 0.248 | 1 | 4 | 0.167–95.756 | 0.392 | 1 | 4192.186 | 0–NA | 0.773 |
|  | Hyperparathyroidism | 1 | 0.002 | 0–NA | 0.794 |  |  |  |  | 1 | 0.005 | 0–NA | 0.808 |
|  | Bone deformity and related symptoms | 1 | 1.461 | 0.242–8.821 | 0.68 | 1 | 0.849 | 0.056–12.915 | 0.906 | 1 | 0.842 | 0.049–14.565 | 0.906 |
|  | Abnormal gait | 1 | 0.001 | 0–NA | 0.8 | 1 | 0.003 | 0–NA | 0.81 | 1 | 0.002 | 0–NA | 0.817 |
|  | Bone pain | 1 | 3.484 | 0.516–23.519 | 0.2 |  |  |  |  | 1 | 6310.94 | 0–NA | 0.665 |
|  | Ectopic ossification and related symptom | 1 | 29.751 | 2.982–296.847 | 0.004* | 1 | 19500.433 | 0–NA | 0.647 | 1 | 4207.293 | 0–NA | 0.689 |
|  | Surgery | 1 | 6.104 | 0.983–37.921 | 0.052 | 1 | 1.85 | 0.172–19.96 | 0.612 | 1 | 3606.252 | 0–NA | 0.7 |
|  | Dental problems | 1 | 3.912 | 0.322–47.509 | 0.284 | 1 | 4 | 0.167–95.756 | 0.392 | 1 | 0.016 | 0–NA | 0.856 |
|  | Height | 19.6 | 1.482 | 0.453–4.846 | 0.516 | 44.5 | 4.823 | 0.31–75.093 | 0.261 | 17.2 | 3.088 | 0.193–49.327 | 0.425 |
|  | Height (Z-score) | 1.38 | 1.254 | 0.428–3.671 | 0.68 | 0.723 | 20.336 | 1.486–278.348 | 0.024* | 1.52 | 0.868 | 0.166–4.553 | 0.867 |
|  | RSS mean | 1.562 | 1.999 | 0.262–15.25 | 0.504 | 1.125 | 0 | 0–NA | 0.828 | 1.125 | 0.91 | 0.004–223.389 | 0.973 |
|  | MAD (L) mean | 22.525 | 0.595 | 0.13–2.721 | 0.503 | 12.75 | 1.707 | 0.344–8.471 | 0.513 | 28.85 | 0.027 | 0–6.481 | 0.197 |
|  | MAD (R) mean | 24.325 | 0.876 | 0.261–2.937 | 0.83 | 17.55 | 0.968 | 0.187–5.024 | 0.97 | 23.5 | 0.849 | 0.101–7.144 | 0.881 |
|  | 6MWT | 91 | 1.016 | 0.39–2.648 | 0.974 | 79 | 1.009 | 0.362–2.815 | 0.987 | 90.25 | 0.457 | 0.036–5.749 | 0.544 |
|  | Grip strength mean | 7 | 0.633 | 0.06–6.649 | 0.703 |  |  |  |  | 7.25 | 0.255 | 0.01–6.394 | 0.406 |
| Total days/year of school non-attendance due to management of XLH | Calcification | 1 | 0.375 | 0.119–1.181 | 0.094 | 1 | 5.459 | 0.219–136.24 | 0.301 | 1 | 0.246 | 0.068–0.889 | 0.032* |
|  | Hyperparathyroidism | 1 | 0.308 | 0.05–1.893 | 0.204 |  |  |  |  | 1 | 0.337 | 0.053–2.139 | 0.249 |
|  | Bone deformity and related symptoms | 1 | 0.404 | 0.139–1.171 | 0.095 | 1 | 0.131 | 0.011–1.536 | 0.106 | 1 | 0.457 | 0.136–1.537 | 0.206 |
|  | Abnormal gait | 1 | 0.311 | 0.08–1.212 | 0.092 | 1 | 0.001 | 0–NA | 0.708 | 1 | 0.426 | 0.102–1.785 | 0.243 |
|  | Bone pain | 1 | 1.292 | 0.313–5.325 | 0.723 |  |  |  |  | 1 | 1.447 | 0.328–6.373 | 0.625 |
|  | Ectopic ossification and related symptom | 1 | 2.043 | 0.621–6.722 | 0.24 | 1 | 6.049 | 0.522–70.122 | 0.15 | 1 | 1.375 | 0.348–5.433 | 0.65 |
|  | Surgery | 1 | 0.412 | 0.133–1.271 | 0.123 | 1 | 0.135 | 0.011–1.611 | 0.113 | 1 | 0.532 | 0.145–1.95 | 0.341 |
|  | Dental problems | 1 | 1.66 | 0.19–14.487 | 0.647 | 1 | 5.459 | 0.219–136.24 | 0.301 | 1 | 0.509 | 0.04–6.397 | 0.601 |
|  | Height | 19.6 | 0.6 | 0.318–1.134 | 0.116 | 44.5 | 0.681 | 0.073–6.372 | 0.736 | 17.2 | 0.56 | 0.254–1.235 | 0.151 |
|  | Height (Z-score) | 1.38 | 0.854 | 0.462–1.58 | 0.615 | 0.723 | 5.218 | 1.009–27 | 0.049* | 1.52 | 0.68 | 0.322–1.437 | 0.312 |
|  | RSS mean | 1.562 | 0.701 | 0.12–4.085 | 0.693 |  |  |  |  | 1.125 | 1.354 | 0.187–9.776 | 0.764 |
|  | MAD (L) mean | 22.525 | 0.876 | 0.214–3.585 | 0.854 | 12.75 | 0.924 | 0.284–3.003 | 0.895 | 28.85 | 0.674 | 0.206–2.201 | 0.513 |
|  | MAD (R) mean | 24.325 | 0.638 | 0.316–1.286 | 0.208 | 17.55 | 0.869 | 0.248–3.049 | 0.827 | 23.5 | 0.568 | 0.192–1.677 | 0.306 |
|  | 6MWT | 91 | 0.88 | 0.494–1.568 | 0.664 | 79 | 0.971 | 0.325–2.903 | 0.958 | 90.25 | 0.953 | 0.422–2.15 | 0.907 |
|  | Grip strength mean | 7 | 3.071 | 0.035–268.461 | 0.623 |  |  |  |  | 7.25 | 4.139 | 0.265–64.576 | 0.311 |
| **Work (adults)** | | **All (*N* = 24)** | | | | **Subgroup analysis** | | | | | | | |
|  |  |  |  |  |  | **Male (*n* = 11)** | | | | **Female (*n*=13)** | | | |
| **Outcome** | **Exposure** | **Diff (IQR)** | **Odds ratio (/IQR)** | **95% confidence interval** | ***P*** | **Diff (IQR)** | **Odds ratio (/IQR)** | **95% confidence interval** | ***P*** | **Diff (IQR)** | **Odds ratio (/IQR)** | **95% confidence interval** | ***P*** |
| Total days/year of work non-attendance due to symptoms/complications of XLH | Renal dysfunction | 1 | 3.392 | 0.228–50.547 | 0.375 | 1 | 0.005 | 0–NA | 0.855 | 1 | 17.083 | 0.456–640.392 | 0.125 |
|  | Hypertension | 1 | 5.367 | 0.407–70.811 | 0.202 | 1 | 1173.664 | 0–NA | 0.801 | 1 | 6.099 | 0.246–151.237 | 0.27 |
|  | Calcification | 1 | 3.462 | 0.269–44.547 | 0.341 | 1 | 1564.885 | 0–NA | 0.777 | 1 | 1.441 | 0.071–29.223 | 0.812 |
|  | Hyperparathyroidism | 1 | 1.075 | 0.083–13.971 | 0.956 | 1 | 0.001 | 0–NA | 0.823 | 1 | 6.099 | 0.246–151.237 | 0.27 |
|  | Bone deformity and related symptoms | 1 | 0.002 | 0–NA | 0.806 | 1 | 0.004 | 0–NA | 0.819 | 1 | 0.004 | 0–NA | 0.813 |
|  | Abnormal gait | 1 | 0.005 | 0–NA | 0.834 | 1 | 0.014 | 0–NA | 0.866 | 1 | 0.013 | 0–NA | 0.826 |
|  | Bone pain | 1 | 1.157 | 0.089–15.109 | 0.911 | 1 | 0.001 | 0–NA | 0.823 | 1 | 6.099 | 0.246–151.237 | 0.27 |
|  | Ectopic ossification and related symptom | 1 | 1.075 | 0.083–13.971 | 0.956 | 1 | 0.001 | 0–NA | 0.823 | 1 | 6.099 | 0.246–151.237 | 0.27 |
|  | Hearing impairment | 1 | 0.005 | 0–NA | 0.834 | 1 | 0.005 | 0–NA | 0.855 |  |  |  |  |
|  | Surgery | 1 | 0.668 | 0.052–8.514 | 0.756 | 1 | 287.678 | 0–NA | 0.786 | 1 | 0.001 | 0–NA | 0.832 |
|  | Dental problems | 1 | 3.392 | 0.228–50.547 | 0.375 | 1 | 0.005 | 0–NA | 0.855 | 1 | 17.083 | 0.456–640.392 | 0.125 |
|  | Height | 10.25 | 1.169 | 0.179–7.651 | 0.871 | 7.7 | 1.527 | 0.109–21.43 | 0.754 | 9.7 | 1.033 | 0.096–11.109 | 0.979 |
|  | Height (Z-score) | 1.488 | 1.307 | 0.377–4.536 | 0.673 | 1.325 | 1.464 | 0.117–18.271 | 0.767 | 1.85 | 1.034 | 0.096–11.126 | 0.978 |
|  | Timed Up and Go Test | 2.64 | 1.425 | 0.886–2.291 | 0.144 | 5.175 | 9724.114 | 0–NA | 0.804 | 2.71 | 0.196 | 0.008–5.05 | 0.325 |
|  | Grip strength mean | 9 | 1.01 | 0.21–4.858 | 0.99 | 7.75 | 0.756 | 0.025–22.818 | 0.872 | 5 | 1.55 | 0.307–7.838 | 0.596 |
| Total days/year of work non-attendance due to management of XLH | Renal dysfunction | 1 | 11.651 | 1.33–102.041 | 0.027* | 1 | 17.63 | 0.716–434.041 | 0.079 | 1 | 10 | 0.317–315.279 | 0.191 |
|  | Hypertension | 1 | 6.738 | 1.24–36.599 | 0.027* | 1 | 43.048 | 1.911–969.873 | 0.018* | 1 | 1.678 | 0.094–29.837 | 0.725 |
|  | Calcification | 1 | 1.543 | 0.334–7.127 | 0.578 | 1 | 3.351 | 0.337–33.319 | 0.302 | 1 | 0.76 | 0.088–6.53 | 0.803 |
|  | Hyperparathyroidism | 1 | 4.44 | 0.812–24.266 | 0.085 | 1 | 9.609 | 0.789–117.039 | 0.076 | 1 | 1.678 | 0.094–29.837 | 0.725 |
|  | Bone deformity and related symptoms | 1 | 0.939 | 0.171–5.166 | 0.942 | 1 | 1 | 0.1–9.957 | 1 | 1 | 0.754 | 0.051–11.079 | 0.837 |
|  | Abnormal gait | 1 | 0.001 | 0–NA | 0.776 | 1 | 0.001 | 0–NA | 0.768 | 1 | 0.002 | 0–NA | 0.781 |
|  | Bone pain | 1 | 2.774 | 0.591–13.015 | 0.196 | 1 | 4.211 | 0.407–43.53 | 0.228 | 1 | 2.644 | 0.222–31.503 | 0.442 |
|  | Ectopic ossification and related symptom | 1 | 1.493 | 0.297–7.51 | 0.627 | 1 | 1.251 | 0.142–11.01 | 0.84 | 1 | 1.678 | 0.094–29.837 | 0.725 |
|  | Hearing impairment | 1 | 1 | 0.073–13.644 | 1 | 1 | 1 | 0.059–16.961 | 1 |  |  |  |  |
|  | Surgery | 1 | 1.102 | 0.246–4.937 | 0.899 | 1 | 0.772 | 0.075–7.962 | 0.828 | 1 | 1.219 | 0.133–11.18 | 0.861 |
|  | Dental problems | 1 | 2.241 | 0.275–18.282 | 0.451 | 1 | 1 | 0.059–16.961 | 1 | 1 | 10 | 0.317–315.279 | 0.191 |
|  | Height | 10.25 | 0.48 | 0.158–1.461 | 0.196 | 7.7 | 0.581 | 0.205–1.646 | 0.307 | 9.7 | 0.451 | 0.072–2.833 | 0.396 |
|  | Height (Z-score) | 1.488 | 0.642 | 0.318–1.299 | 0.218 | 1.325 | 0.575 | 0.205–1.613 | 0.293 | 1.85 | 0.451 | 0.072–2.836 | 0.396 |
|  | Timed Up and Go Test | 2.64 | 1.143 | 0.797–1.638 | 0.467 | 5.175 | 1.161 | 0.51–2.643 | 0.722 | 2.71 | 2.32 | 0.368–14.636 | 0.371 |
|  | Grip strength mean | 9 | 0.869 | 0.326–2.319 | 0.779 | 7.75 | 0.422 | 0.067–2.64 | 0.356 | 5 | 0.818 | 0.323–2.073 | 0.672 |

/IQRs represent the odds ratio corresponding to a change in the explanatory variable of IQR.

**P*-value <0.05

6MWT = 6-minute walk test; IQR = interquartile range; L = left; MAD = mechanical axis deviation; NA = not applicable; R = right; RSS = Rickets severity score; XLH = X-linked hypophosphatemic rickets/osteomalacia.

**Supplementary Table S3.** Relationships between school/work status and SF-10, FPS-R, BPI, and WOMAC (full data)

| **School (children)** | | | **All (*N* = 40)** | | | | | | | | **Subgroup analysis** | | | | | | | | | | |
| --- | --- | --- | --- | --- | --- | --- | --- | --- | --- | --- | --- | --- | --- | --- | --- | --- | --- | --- | --- | --- | --- |
|  |  |  |  |  |  |  |  |  |  |  | **Male (*n* = 11)** | | | | | **Female (*n* = 29)** | | | | | |
| **Outcome** | | **Exposure** | **Diff (IQR)** | **Odds ratio (/IQR)** | | **95% confidence interval** | | | ***P*** | | **Diff (IQR)** | **Odds ratio (/IQR)** | **95% confidence interval** | ***P*** | | **Diff (IQR)** | **Odds ratio (/IQR)** | | **95% confidence interval** | | ***P*** |
| Total days/year of school non-attendance due to symptoms/ complications of XLH | | SF-10: PHS score | 13.86 | 0.067 | | 0.008–0.562 | | | 0.013* | | 11.81 | 0.001 | 0–1.415 | 0.063 | | 10.958 | 0.469 | | 0.057–3.855 | | 0.481 |
|  |  | SF-10: PSS score | 8.02 | 0.162 | | 0.036–0.724 | | | 0.017* | | 2.675 | 0.508 | 0.267–0.963 | 0.038* | | 11.36 | 0.46 | | 0.011–19.893 | | 0.686 |
|  |  | FPS-R | 2 | 3.967 | | 0.229–68.607 | | | 0.343 | | 1 | 2.017 | 0.177–23.021 | 0.572 | | 2 | 15.758 | | 0.133–NA | | 0.257 |
| Total days/year of school non-attendance due to management of XLH | | SF-10: PHS score | 13.86 | 0.476 | | 0.193–1.176 | | | 0.108 | | 11.81 | 0.057 | 0.005–0.61 | 0.018* | | 10.958 | 0.974 | | 0.358–2.654 | | 0.959 |
|  |  | SF-10: PSS score | 8.02 | 0.605 | | 0.29–1.262 | | | 0.18 | | 2.675 | 0.641 | 0.381–1.078 | 0.094 | | 11.36 | 0.796 | | 0.233–2.726 | | 0.717 |
|  |  | FPS-R | 2 | 2.453 | | 0.361–16.685 | | | 0.359 | | 1 | 3.75 | 0.328–42.868 | 0.288 | | 2 | 1.463 | | 0.175–12.216 | | 0.725 |
| **Work(adults)** | | | **All (*N* = 23)** | | | | | | | **Subgroup analysis** | | | | | | | | | | | |
|  |  |  |  |  |  |  |  |  |  | **Male (*n* = 10)** | | | | | **Female (*n* = 13)** | | | | | | |
| **Outcome** | **Exposure** | | **Diff (IQR)** | | **Odds ratio (/IQR)** | | **95% confidence interval** | ***P*** | | **Diff (IQR)** | | **Odds ratio (/IQR)** | **95% confidence interval** | ***P*** | **Diff (IQR)** | | **Odds ratio (/IQR)** | | **95% confidence interval** | | ***P*** |
| Total days/year of work non-attendance due to symptoms/ complications of XLH | BPI-pain severity (worst) | | 5 | | 43.195 | | 0.813–NA | 0.063 | | 1.75 | | 3044.583 | 0–NA | 0.759 | 6 | | 13.85 | | 0.178–NA | | 0.237 |
|  | BPI-pain severity (least) | | 2 | | 1.588 | | 0.266–9.498 | 0.612 | | 1.75 | | 6.927 | 0.193–249.157 | 0.29 | 3 | | 0.782 | | 0.01–64.349 | | 0.913 |
|  | BPI-pain severity (average) | | 4.75 | | 156.522 | | 0.401–NA | 0.097 | | 2.75 | | 240061721.3 | 0–NA | 0.767 | 5 | | 47.946 | | 0.196–NA | | 0.168 |
|  | BPI-pain severity (now) | | 2 | | 3.138 | | 0.979–10.06 | 0.054 | | 2 | | 2132460.356 | 0–NA | 0.789 | 2.5 | | 2.358 | | 0.421–13.208 | | 0.329 |
|  | BPI-pain interference | | 2.964 | | 7.466 | | 1.335–41.763 | 0.022* | | 2.964 | | 54675.676 | 0–NA | 0.84 | 1.607 | | 2.286 | | 0.823–6.355 | | 0.113 |
|  | WOMAC Pain | | 17.5 | | 2.499 | | 0.861–7.257 | 0.092 | | 12.5 | | 153230521.2 | 0–NA | 0.807 | 15 | | 1.575 | | 0.437–5.676 | | 0.487 |
|  | WOMAC Stiffness | | 25 | | 3.85 | | 0.973–15.226 | 0.055 | | 34.375 | | 804753920.4 | 0–NA | 0.803 | 25 | | 3.882 | | 0.333–45.311 | | 0.279 |
|  | WOMAC Physical function | | 12.5 | | 2.53 | | 1.069–5.985 | 0.035 | | 11.397 | | 356.144 | 0–NA | 0.751 | 7.353 | | 2.65 | | 0.703–9.981 | | 0.15 |
| Total days/year of work non-attendance due to management of XLH | BPI-pain severity (worst) | | 5 | | 1.66 | | 0.411–6.702 | 0.477 | | 1.75 | | 0.956 | 0.438–2.087 | 0.91 | 6 | | 2.812 | | 0.284–27.808 | | 0.376 |
|  | BPI-Pain Severity (least) | | 2 | | 1.447 | | 0.433–4.839 | 0.548 | | 1.75 | | 1.035 | 0.224–4.781 | 0.964 | 3 | | 1.547 | | 0.108–22.213 | | 0.748 |
|  | BPI-pain severity (average) | | 4.75 | | 7.165 | | 1.189–43.183 | 0.032* | | 2.75 | | 3.091 | 0.458–20.838 | 0.247 | 5 | | 3.974 | | 0.374–42.219 | | 0.252 |
|  | BPI-pain severity (now) | | 2 | | 1.13 | | 0.537–2.377 | 0.748 | | 2 | | 0.808 | 0.269–2.421 | 0.703 | 2.5 | | 1.858 | | 0.426–8.11 | | 0.41 |
|  | BPI-pain interference | | 2.964 | | 1.552 | | 0.615–3.918 | 0.352 | | 2.964 | | 0.928 | 0.256–3.364 | 0.91 | 1.607 | | 1.977 | | 0.85–4.598 | | 0.114 |
|  | WOMAC Pain | | 17.5 | | 0.91 | | 0.423–1.959 | 0.81 | | 12.5 | | 0.772 | 0.355–1.677 | 0.513 | 15 | | 1.099 | | 0.371–3.256 | | 0.865 |
|  | WOMAC Stiffness | | 25 | | 1.104 | | 0.479–2.543 | 0.817 | | 34.375 | | 0.838 | 0.198–3.552 | 0.811 | 25 | | 1.665 | | 0.271–10.234 | | 0.582 |
|  | WOMAC Physical function | | 12.5 | | 0.994 | | 0.537–1.84 | 0.985 | | 11.397 | | 0.756 | 0.358–1.596 | 0.463 | 7.353 | | 1.908 | | 0.663–5.493 | | 0.231 |
| **School or work (adults)** | | | **All (*N* = 31)** | | | | | | | **Subgroup analysis** | | | | | | | | | | | |
|  |  |  |  |  |  |  |  |  |  | **Male (*n* = 11)** | | | | | **Female (*n* = 20)** | | | | | | |
| **Outcome** | **Exposure** | | **Diff (IQR)** | | **Odds ratio (/IQR)** | | **95% confidence interval** | ***P*** | | **Diff (IQR)** | | **Odds ratio (/IQR)** | **95% confidence interval** | ***P*** | **Diff (IQR)** | | | **Odds ratio (/IQR)** | | **95% confidence interval** | ***P*** |
| Total days/year of school/work non-attendance due to symptoms/ complications of XLH | BPI-pain severity (worst) | | 5 | | 16.291 | | 1.392–190.693 | 0.026* | | 2 | | 7437.639 | 0–NA | 0.742 | 5 | | | 6.273 | | 0.526–74.775 | 0.146 |
|  | BPI-pain severity (least) | | 1.25 | | 1.264 | | 0.446–3.584 | 0.66 | | 2 | | 9.648 | 0.174–534.79 | 0.269 | 3 | | | 0.93 | | 0.015–57.304 | 0.973 |
|  | BPI-pain severity (average) | | 4 | | 11.41 | | 1.111–117.14 | 0.04* | | 3 | | 854746593.3 | 0–NA | 0.753 | 4 | | | 7.254 | | 0.724–72.688 | 0.092 |
|  | BPI-pain severity (now) | | 2 | | 2.717 | | 1.023–7.215 | 0.045* | | 2 | | 1487272.124 | 0–NA | 0.777 | 1 | | | 1.365 | | 0.744–2.505 | 0.316 |
|  | BPI-pain interference | | 2.893 | | 4.331 | | 1.197–15.675 | 0.026* | | 2.929 | | 41826.961 | 0–NA | 0.833 | 2.714 | | | 2.485 | | 0.609–10.143 | 0.205 |
|  | WOMAC Pain | | 20 | | 2.497 | | 0.827–7.539 | 0.104 | | 15 | | 1303542850 | 0–NA | 0.766 | 20 | | | 1.715 | | 0.434–6.785 | 0.442 |
|  | WOMAC Stiffness | | 25 | | 3.735 | | 1.132–12.33 | 0.031* | | 31.25 | | 86044417.24 | 0–NA | 0.792 | 25 | | | 4.122 | | 0.508–33.465 | 0.185 |
|  | WOMAC Physical function | | 14.338 | | 3.044 | | 1.195–7.75 | 0.02* | | 11.765 | | 375.012 | 0–NA | 0.737 | 14.706 | | | 3.607 | | 0.556–23.404 | 0.179 |
| Total days/year of school/work non-attendance due to management of XLH | BPI-pain severity (worst) | | 5 | | 1.717 | | 0.379–7.788 | 0.483 | | 2 | | 0.869 | 0.384–1.967 | 0.736 | 5 | | | 2.704 | | 0.316–23.16 | 0.364 |
|  | BPI-pain severity (least) | | 1.25 | | 1.198 | | 0.557–2.575 | 0.645 | | 2 | | 0.882 | 0.173–4.487 | 0.88 | 3 | | | 1.322 | | 0.091–19.303 | 0.838 |
|  | BPI-pain severity (average) | | 4 | | 4.418 | | 0.915–21.338 | 0.064 | | 3 | | 2.173 | 0.378–12.485 | 0.384 | 4 | | | 2.79 | | 0.337–23.105 | 0.341 |
|  | BPI-pain severity (now) | | 2 | | 1.181 | | 0.531–2.624 | 0.683 | | 2 | | 0.739 | 0.258–2.116 | 0.573 | 1 | | | 1.216 | | 0.678–2.182 | 0.511 |
|  | BPI-pain interference | | 2.893 | | 1.495 | | 0.591–3.783 | 0.395 | | 2.929 | | 0.82 | 0.242–2.776 | 0.75 | 2.714 | | | 2.343 | | 0.653–8.409 | 0.192 |
|  | WOMAC Pain | | 20 | | 0.888 | | 0.407–1.936 | 0.765 | | 15 | | 0.676 | 0.278–1.647 | 0.389 | 20 | | | 1.141 | | 0.363–3.583 | 0.822 |
|  | WOMAC Stiffness | | 25 | | 0.918 | | 0.421–2 | 0.829 | | 31.25 | | 0.738 | 0.212–2.571 | 0.633 | 25 | | | 0.869 | | 0.183–4.112 | 0.859 |
|  | WOMAC Physical function | | 14.338 | | 0.966 | | 0.495–1.888 | 0.92 | | 11.765 | | 0.698 | 0.326–1.493 | 0.354 | 14.706 | | | 2.358 | | 0.534–10.417 | 0.258 |

/IQRs represent the odds ratio corresponding to a change in the explanatory variable of IQR.

**P-*value <0.05

BPI = brief pain inventory; FPS-R = revised faces pain scale; IQR = interquartile range; NA = not applicable; PHS = physical; PSS = psychosocial; SF-10 = 10-item short-form health survey; WOMAC = Western Ontario and McMaster Universities osteoarthritis index; XLH = X-linked hypophosphatemic rickets/osteomalacia.

**Supplementary Table S4.** Normative values of serum phosphorus, alkaline phosphatase, calcium, and creatinine by age

| **Parameter, Unit** | **Age** | **Normal range** | |
| --- | --- | --- | --- |
|  |  | **Male** | **Female** |
| Serum phosphorus, mg/dL | 0 month | 5.0–7.7 | 5.0–7.7 |
|  | 1 month | 4.8–7.5 | 4.8–7.5 |
|  | 3 months | 4.5–7.1 | 4.5–7.1 |
|  | 6 months | 4.2–6.7 | 4.2–6.7 |
|  | 1 year | 3.9–6.2 | 3.9–6.2 |
|  | 2 years | 3.8–6.0 | 3.8–6.0 |
|  | 3 years | 3.8–5.9 | 3.8–5.9 |
|  | 6 years | 3.9–5.8 | 3.9–5.8 |
|  | 12 years | 3.6–5.8 | 3.6–5.8 |
|  | 15 years | 3.2–5.5 | 3.2–5.5 |
|  | 20 years | 2.8–4.7 | 2.8–4.7 |
|  | >20 years | 2.4–4.3 | 2.4–4.3 |
| Alkaline phosphatase (IFCC)*, IU/L | 0 month | 185.5–563.5 | 185.5–563.5 |
|  | 1 month | 178.5–567 | 178.5–567 |
|  | 3 months | 168–567 | 168–567 |
|  | 6 months | 147–553 | 147–553 |
|  | 1 year | 138.25–468.65 | 138.25–451.15 |
|  | 2 years | 143.5–437.5 | 143.5–402.5 |
|  | 3 years | 147–420 | 147–395.5 |
|  | 6 years | 154–430.5 | 161–437.5 |
|  | 12 years | 159.25–525 | 105–483 |
|  | 15 years | 94.5–420 | 54.25–315 |
|  | 20 years | 52.5–143.5 | 42–119 |
| Serum calcium, mg/dL | 0 month | 9.0–11.0 | 9.0–11.0 |
|  | 1 month | 9.0–11.0 | 9.0–11.0 |
|  | 3 months | 9.0–11.0 | 9.0–11.0 |
|  | 6 months | 9.0–11.0 | 9.0–11.0 |
|  | 1 year | 8.8–10.6 | 8.8–10.6 |
|  | 2 years | 8.8–10.5 | 8.8–10.5 |
|  | 3 years | 8.8–10.3 | 8.8–10.3 |
|  | 6 years | 8.7–10.2 | 8.7–10.2 |
|  | 12 years | 8.7–10.1 | 8.7–10.1 |
|  | 15 years | 8.7–10.0 | 8.7–10.0 |
|  | 20 years | 8.7–10.0 | 8.7–10.0 |
| Serum creatinine, 2.5, 50, 97.5 percentile, mg/mL | 3–5 months | 0.12, 0.2, 0.27 | 0.12, 0.2, 0.27 |
|  | 6–8 months | 0.13, 0.21, .33 | 0.13, 0.21, .33 |
|  | 9–11 months | 0.14, 0.23, 0.35 | 0.14, 0.23, 0.35 |
|  | 1 year | 0.14, 0.23, 0.35 | 0.14, 0.23, 0.35 |
|  | 2 years | 0.17, 0.24, 0.45 | 0.17, 0.24, 0.45 |
|  | 3 years | 0.2, 0.27, 0.39 | 0.2, 0.27, 0.39 |
|  | 4 years | 0.2, 0.3, 0.41 | 0.2, 0.3, 0.41 |
|  | 5 years | 0.25, 0.34, 0.45 | 0.25, 0.34, 0.45 |
|  | 6 years | 0.25, 0.34, 0.48 | 0.25, 0.34, 0.48 |
|  | 7 years | 0.28, 0.37, 0.5 | 0.28, 0.37, 0.5 |
|  | 8 years | 0.27, 0.4, 0.53 | 0.27, 0.4, 0.53 |
|  | 9 years | 0.3, 0.41, 0.55 | 0.3, 0.41, 0.55 |
|  | 10 years | 0.3, 0.4, 0.61 | 0.3, 0.4, 0.61 |
|  | 11 years | 0.34, 0.45, 0.61 | 0.34, 0.45, 0.61 |
|  | 12 years | 0.39, 0.53, 0.62 | 0.39, 0.52, 0.69 |
|  | 13 years | 0.4, 0.59, 0.81 | 0.4, 0.53, 0.7 |
|  | 14 years | 0.54, 0.65, 1.05 | 0.46, 0.58, 0.72 |
|  | 15 years | 0.47, 0.68, 0.93 | 0.47, 0.56, 0.72 |

*Normative values of serum alkaline phosphatase were converted from the Japan Society of Clinical Chemistry (JSCC) to the International Federation of Clinical Chemistry (IFCC). IFCC estimated as approximately 0.35 × the JSCC values [52]
